# Effects of Germination on Protein, γ-Aminobutyric Acid, Phenolic Acids, and Antioxidant Capacity in Wheat

**DOI:** 10.3390/molecules23092244

**Published:** 2018-09-03

**Authors:** Mi Jeong Kim, Han Sub Kwak, Sang Sook Kim

**Affiliations:** 1 Research Group of Food Processing, Korea Food Research Institute, Jeollabuk-do 55465, Korea; mj78.kim@gmail.com (M.J.K.); hskwak@kfri.re.kr (H.S.K.); 2 Department of Food and Nutrition, Changwon National University, Changwon 51140, Korea

**Keywords:** wheat, germination, antioxidant capacity, phenolic acid, two-dimensional gel electrophoresis (2-DE)

## Abstract

Germinated wheat is a food material with potential health benefits due to its high phenolic and antioxidant content, but the reason why germination increases this content is unclear. The aim of this study was to investigate the relationships between protein changes (determined by two-dimensional gel electrophoresis (2-DE)), phenolics, γ-aminobutyric acid (GABA) levels, and antioxidant capacity of wheat germinated for various periods (24, 48, 72, and 96 h) compared to control. Each phenolic acid tended to increase with increasing germination time. The GABA content was highest (39.98 mg/100 g dwb) after 96 h of germination. The total oxygen radical absorbance capacity (ORAC) was 1.97 times higher after 96 h than in ungerminated seeds. Fifteen proteins, among 82 proteins separated by 2-DE, were highly related with ORAC and were identified by peptide mass fingerprinting (PMS). The PMS revealed strong expression of granule bound starch synthase (GBSS) and glutathione S-transferase (GSTF) after 96 h of germination. Overall, the ORAC at 96 h exhibited a close relationship with the levels of phenolic acids, GABA, and proteins such as GBSS and GSTF. In conclusion, these findings add to the existing knowledge of wheat protein changes and their relationship to the antioxidant properties of germinating wheat seeds.

## 1. Introduction

Wheat (*Triticum aestivum* L.), one of the three major grains, is harvested worldwide because it grows well in dry climates. Over the past two centuries, wheat used for human consumption has mostly been refined before its utilization as a food or an ingredient, but consumption of whole wheat has recently been recommended due to its health benefits [1]. Whole wheat products are rich in dietary fiber, minerals, vitamins, and phytochemicals, and their consumption is associated with reduced occurrences of certain diseases, such as cancer, diabetes, and coronary cardiovascular disease [2]. Consumers today are becoming more health conscious, so researchers are striving more than ever to develop healthy cereal products, including whole wheat products. 

One method to improve the nutritional quality of cereal products is to germinate the grain [3,4,5,6]. The germination process occurs under suitable conditions of moisture and temperature [7] and activates endogenous enzymes responsible for the breakdown of starch and protein into small molecules such as sucrose, maltose, glucose, and amino acids [8]. The increased activation of the starch-degrading α-amylase and various proteases reduces total dry matter [9], while the significant increases in free amino acids, soluble dietary fiber, γ-aminobutyric acid (GABA), vitamins, minerals, and phytochemicals enhance the potential health benefits of germinated cereals [4,5,8,10]. 

Only a few studies have investigated the turnover of phytochemicals such as individual phenolic acids and antioxidants in germinated wheat [4,11,12]. The major phenolic compound found in two Canadian wheat varieties germinated for 2 days was syringic acid [12]. Another recent study reported that the content of free and bound phenolics of Chinese wheat varieties significantly increased during germination and contributed to enhanced antioxidant capacity [11]. However, no research has yet determined why the germination process increases the levels of phenolic compounds and antioxidants in wheat. 

Understanding the physiological and biochemical characteristics of wheat germination may require knowledge of the germination-induced alterations in protein profiles, which can be obtained by methods such as two-dimensional gel electrophoresis (2-DE) [13]. One previous study reported that different proteins expressed during wheat germination and visualized on 2-DE gels were related to different metabolic pathways, such as those related to the production of amino acids, carbohydrates, nucleic acids, and stress-related proteins, thereby providing information regarding molecular mechanisms related to germination [14]. However, no relationship has yet been established between these protein changes and the germination-related production of compounds or biological activities that are beneficial to human health. Therefore, the objective of this study was to investigate the relationships between protein changes observed by 2-DE analysis and increases in antioxidant properties in wheat grains sampled at various time points (24, 48, 72, and 96 h) during germination. In addition, the proteins that showed high correlation with antioxidant capacity were identified by peptide mass fingerprinting (PMF) and related to the changes in individual phenolic acids and GABA levels in ungerminated and germinated wheat grains. 

## 2. Results and Discussion

### 2.1. Phenolic Acid Compositions of Germinated Wheat

Individual phenolic acids occurring in ungerminated control grains and wheat grains at different times (24, 48, 72, and 96 h) during germination are listed in Table 1. The free (alcohol soluble fraction) and bound (insoluble fraction) phenolic extracts contained gallic, 4-hydroxybenzoic, *p*-coumaric, vanillic, syringic, ferulic, and caffeic acids. Ferulic acid was the predominant phenolic acid identified in all five wheat samples, in agreement with similar findings in two previous studies [11,12]. As shown in Table 1, the amounts of free and bound ferulic acids significantly differed (both *p* < 0.001) in the five samples, ranging from 1.04 to 1.62 mg/100 g dry weight basis (dwb) and from 40.19 to 63.13 mg/100 g dwb, respectively. In particular, the levels of bound ferulic acid were 1.5 times higher in grains germinated for 96 h (63.13 mg/100 g dwb) than in ungerminated control grains (40.19 mg/100 g dwb). The level of bound vanillic acid also showed a significant increase with germination time, as this level was 1.51 mg/100 g dwb in the ungerminated grains and 19.43 mg/100 g dwb in grains germinated for 96 h, implying that the germination process effectively increased the phenolic acid content of the wheat grains. These results also aligned with the findings of Chen et al. [11], who found similar changes in the phenolic acid compositions of different Chinese wheat varieties during germination [11]. 

Table 1 also shows that germination significantly increased the levels of vanillic, gallic, caffeic, syringic, and 4-hydroxybenzoic acids in the free fractions, confirming previous reports on phenolic acid profiles of germinated Canada Western Red Spring (CWRS) wheat [12]. The levels of free 4-hydroxybenzoic, vanillic, caffeic, syringic, and gallic acids were, respectively, 24.7, 23.2, 21.4, 14.2, and 8.7 times higher in wheat samples germinated for 96 h than in the ungerminated controls. These increases in phenolic acids during germination might be explained by lignin synthesis during germination [11], as well as by breakdown of lignin and other cell wall polymers as reported in another study [15]. The germination process might induce the synthesis of phenolic compounds [6], and a few studies have reported a relationship between the increased phenolic contents and antioxidant activity observed during seed germination and the expression of phenylalanine ammonia-lyase and glutathione S transferase [16,17]. 

### 2.2. γ-Aminobutyric Acid (GABA) Content of Germinated Wheat Grains

GABA is the main inhibitory neurotransmitter and induces anxiety-calming effects in the mammalian central nervous system; thus, its consumption in foods might assist people suffering from neurological disorders [4]. The GABA contents were significantly different among the wheat samples investigated here (*p* < 0.001), with significant increases observed with germination time when compared with the ungerminated control. As shown in Table 1, the GABA content in the ungerminated control was only 4.5 mg/100 g dwb while the content increased significantly to 39.98 mg/100 g dwb, or almost 9-fold that of the control, after germination for 96 h. A similar significant increase in GABA during germination has been reported previously for other cereals, including wheat [4], barnyard millet [5], and buckwheat [18]. The GABA content (39.98 mg/100 g dwb) of wheat after germination for 96 h in the present study was higher than the content of 24.93 mg/100 g dwb reported by Ohm et al. [14] in wheat germinated for 5 days [4]. Conversely, buckwheat germinated for 4 days contained more GABA than the germinated wheat samples tested in the present study. GABA formation has been attributed to the decarboxylation of l-glutamate due to activation of the glutamate decarboxylase enzyme during germination [19]. Thus, exploitation of the germination process might be an effective means to enhance the GABA content in cereals intended for human consumption; indeed, numerous studies have attempted to utilize germinated wheat in bakery products as a source of GABA [19,20].

### 2.3. Oxygen Radical Absorbance Capacity (ORAC) of Germinated Wheat Grains

**Figure 1:**
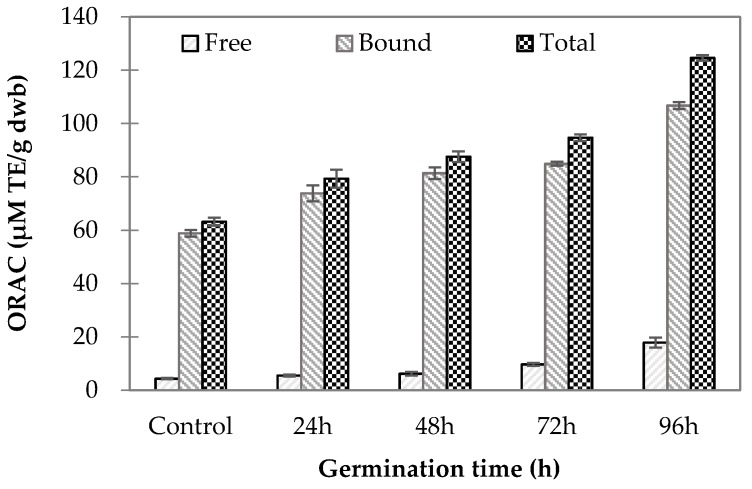
Oxygen radical absorbance capacity (ORAC) of wheat grain samples at various germination times compared to ungerminated control grains. (Lower case letters indicate statistically significant differences in free phenolic content; italicized lower case letters mean statistically significant differences in bound phenolic content; upper case letters mean statistically significant differences in total phenolic content).

The ORAC values for germinated wheat samples are shown in Figure 1. The free and bound ORAC values were significantly different among the samples (*p* < 0.001) and ranged from 4.34 to 17.86 µM Trolox equivalents (TE)/g dwb and 58.84–106.76 µM TE/g dwb, respectively. Overall, the values were significantly higher for bound ORAC than for free ORAC in all samples tested. These results are in agreement with the study by Van Hung et al. [2], who found a higher antioxidant capacity for the bound phenolic extracts than for the free phenolic extracts of both ungerminated and germinated high-amylose wheats [2]. In the present study, ferulic acid was the predominant phenolic among those identified in the wheat grains. The higher bound ORAC values might reflect higher amounts of bound versus free ferulic acid in the extracts. The increase in bound ferulic acids with germination time might be a result of increased amylolytic and proteolytic enzyme activities, as discussed by Xu et al. [6], who reported softening of the kernel structure by these enzymes. This softening could aid in the liberation of some bound forms of phenolic acids [6]. 

The ratio of antioxidant capacity increased with germination time in this study, as the free (17.86 µM TE/g dwb) and bound (106.76 µM TE/g dwb) ORAC values were, respectively, 4.11 and 1.81 times higher in wheat germinated for 96 h than in control ungerminated grains (free ORAC: 4.34 µM TE/g dwb; bound ORAC: 58.84 µM TE/g dwb), indicating a greater increase in antioxidant capacity in the soluble fraction than in the bound fraction during germination. In the present study, the levels of free 4-hydroxybenzoic, caffeic, and syringic acids were markedly increased as germination progressed. Thus, these three phenolic acids might contribute to the observed increases in antioxidant capacity due to free phenolic compounds in germinated wheat samples. The increase in antioxidant capacity in germinated wheat could be attributed to increases in GABA content during germination. GABA is a free modified amino acid that could help in scavenging free radicals [21]. 

### 2.4. Wheat Grain 2-DE Protein Patterns during Germination

**Figure 2:**
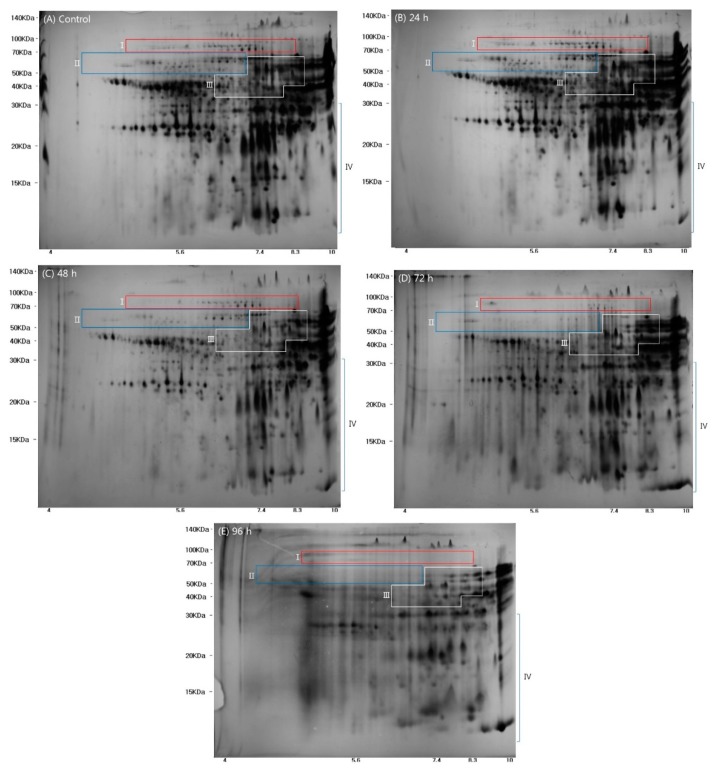
Images of proteins separated after two dimensional gel electrophoresis of ungerminated control wheat grains and wheat grain samples germinated for various times (24, 48, 72, and 96 h) control. (Regions I, II, III, and IV represent (I) high molecular weight glutenin subunit, (II) ω-gliadins, (III) α/β, and γ gliadins and the low molecular weight glutenin subunit; and (IV) nonstorage proteins).

The proteins extracted from wheat grains displayed distinct expression profiles on the 2-D gels, as shown in Figure 2. These results confirmed that the molecular weight (Mw) and isoelectric point (pI) of proteins were 10–140 kDa and 4–10 pI, respectively, in agreement with a previous study [22]. Comparison of the protein spots from the five wheat samples identified 82 spots with at least 1.5-fold differences in intensity, and these were selected for further analysis. 

The selected spot proteins (SSPs) from the five wheat samples are marked with red numbers and green circles in Supplementary Figure S1, and a heatmap showing the relative intensity of the SSPs is presented in Supplementary Figure S2. Based on previous studies [22,23,24], the wheat proteins separated by 2-DE were divided into the following four main regions on the gels: (I) high molecular weight-glutenin subunit (HMW-GS); (II) ω-gliadins; (III) α/β and γ gliadins and low molecular weight glutenin subunit (LMW-GS); and, (IV) nonstorage proteins. The SSPs in the control and germinated wheat samples are shown in Figure 2 and Supplementary Figure S2. The known wheat storage proteins, glutenin (HMW-GS and LMW-GS) and gliadin (α/β, γ, and ω), have been reported to affect dough viscosity and extensibility when making wheat flour products [22]. As shown in Figure 2, expression of proteins in regions I, II, and III gradually declined as germination time increased. These results could be partly explained by the findings of a previous study that reported hydrolysis and metabolism of the storage proteins in the endosperm of wheat by hydrolytic enzymes [14]. Thus, the storage proteins serve as an energy source during germination, resulting in decreases in the amounts of γ-gliadin, α-gliadin, gliadin/avenin-like seed proteins, and avenin-like proteins [14].

### 2.5. Correlation between Protein Spots and Antioxidant Properties in Germinated Wheat Samples

**Figure 3:**
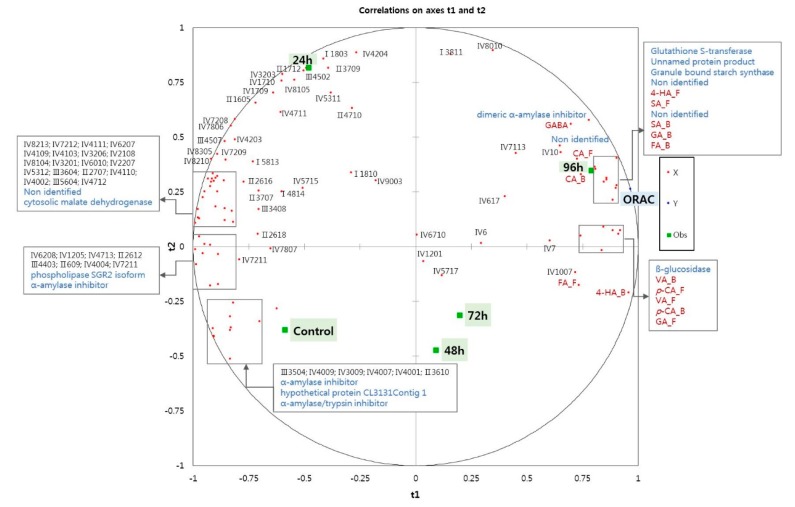
Correlation map of 82 selected protein spots detected on 2DE-gels, phenolic composition, GABA levels (red dot), and ORAC values (blue dot) in ungerminated control and germinated wheat grains after various germination times (24, 48, 72, and 96 h). (CA_F: free caffeic acid; CA_B: bound caffeic acid; FA_F: free ferulic acid; FA_B: bound ferulic acid; SA_F: free syringic acid; SA_B: bound syringic acid; GA_F: free gallic acid; GA_B: bound gallic acid; 4-HA_F: free 4-hydroxybenzoic acid; 4-HA_B: bound hydroxybenzoic acid; VA_F: free vanillic acid; VA_B: bound vanillic acid; *p*-CA_F: free *p*-coumaric acid; *p*-CA_B: bound *p*-coumaric acid. Regions I, II, III, and IV represent (I) high molecular weight-glutenin subunit, (II) ω-gliadins, (III) α/β and γ gliadins and low molecular weight glutenin subunit; (IV) nonstorage proteins. Numbers refer to the protein spot numbers presented as red circles in Supplementary Figure S1).

Figure 3 shows a correlation map generated by the PLS regression analysis of the protein spots selected after 2-DE analysis, and the GABA levels, phenolic acid compositions, and antioxidant capacity (ORAC value) of the five wheat grain samples. The ungerminated wheat (control) was loaded negatively on the t1 and t2 axes, whereas the sample germinated for 96 h was located positively on the t1 and t2 axes, indicating a closed relationship between the longer germination time and antioxidant properties. Previous studies on the proteome of germinated wheat have identified proteins that function in seed development and stress responses, as well as differences between the embryo and endosperm [14,25]. A recent study that investigated the relationships between proteins related to anthocyanins, phenolic compounds, and total antioxidant activity in ungerminated and germinated rice grains also reported an upregulation of isoflavone-7-*O*-methyltransferase, phenylalanine ammonia-lyase (PAL), and glutathione S-transferase (GSTF) during germination [17]. However, proteins related to antioxidant capacity in germinated wheats have not yet been studied using peptide mass fingerprinting (PMF). Thus, fifteen proteins were selected for further analysis in the present study, based on *r* ≥ ±0.7 in the correlation map shown in Figure 3. 

The proteins identified by PMF are shown in Table 2. The relative expressions of alpha-amylase inhibitor, alpha-amylase/trypsin inhibitor, phospholipase SGR2 isoform, and hypothetical protein CL3131Contig 1 substantially decreased as germination progressed (Table 2), in agreement with the findings of Mak et al. [25], who reported a decreasing abundance of these proteins during germination [25]. Contrary to the findings of Mak et al. [25], however, the expression of malate dehydrogenase protein decreased during germination in the present study. In addition, the expressions of granule bound starch synthase (GBSS), dimeric alpha-amylase inhibitor, glutathione S-transferase (GSTF), and β-glucosidase were increased as germination progressed (Table 2). Figure 3 shows that GSTF and GBSS expressions were highly correlated with the ORAC value and these proteins were strongly expressed in the 96 h germinated wheat sample. The levels of free 4-hydroxybenzoic acid, syringic acid (free and bound), bound gallic acid, bound ferulic acid, and caffeic acid (free and bound) were closely related to the ORAC value, suggesting that the levels of these phenolic acids might be increased by the activities of proteins such as GSFT and GBSS. The GSTF enzyme is linked to the biosynthesis of phenolic compounds and plays a role in redox regulation [14]. Other redox regulation proteins, such as superoxide dismutase, GSFT, and peroxidases, can scavenge free radicals, indicating a function as antioxidant enzymes. In the wheat grain, therefore, GSFT and GBSS activity might be related to the biosynthesis of phenolic compounds so that these enzymes contribute to the antioxidant capacity of germinating wheat grains. As shown by the correlation map, GABA was closely located with the dimeric α-amylase inhibitor found in malted barley [26]. Vanillic acid (free and bound), *p*-coumaric acid (free and bound), and free vanillic acid were closely located with ß-glucosidase, the key enzyme responsible for cellulose degradation, a process that could liberate phenolic acids from plant cell walls [27,28]. Although the relationship between few proteins and wheat antioxidant capacity is explained by statistical speculation, the GBSS, GSTF, and β-glucosidase increased by germination might be linked with antioxidant capacity and phenolic compositions in germinated wheat. In the future, an experiment such as proteomic analysis will be needed to confirm whether those proteins affect the antioxidation of germinated wheat.

## 3. Materials and Methods

### 3.1. Materials 

#### 3.1.1. Wheat Sample and Germination Procedure

The wheat (*T. aestivum* L. cv. Keumkang) grains used in this study were harvested in Iksan (Jeollabuk-Do, Korea, geographic coordinates: 35°56′ N, 126°53′ E) in 2015. Approximately 1 kg of wheat was washed and then soaked in tap water at a ratio of 1 part wheat to 5 parts water for 6 h at room temperature. The water was then drained off and the wheat grains were germinated at 25 °C for different times (24, 48, 72, and 96 h). 

**Figure 4:**
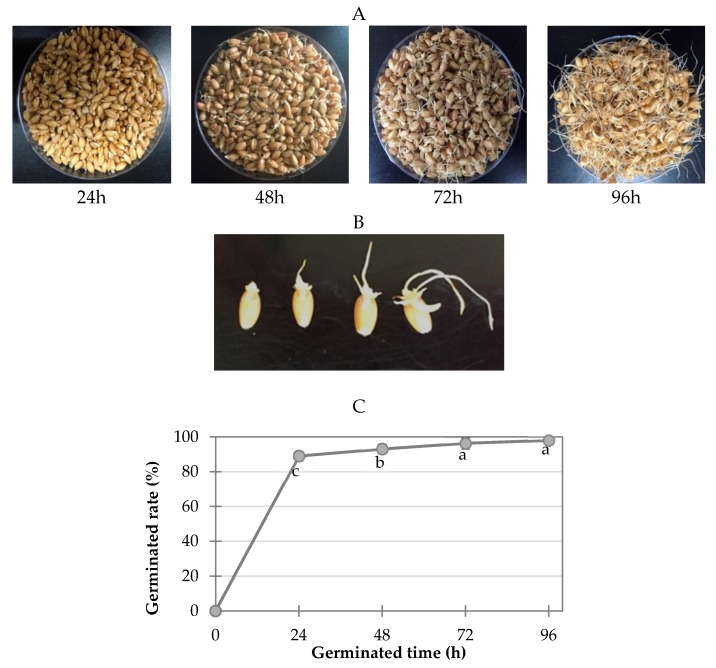
Wheat samples germinated for various times (24 h, 48 h, 72 h, and 96 h; (**A**,**B**)) and the germination rate (**C**) at each germination time point.

The appearance of the wheat samples germinated at various times and the germination rates at each time point are shown in Figure 4. One hundred seeds were randomly removed from each group for each replication and three replications were conducted. Seeds were considered germinated when the radicle had emerged approximately 1 mm or more. The germination rate, calculated as the percentage of germinated kernels, was greater than 90% (Figure 1). The germinated wheat samples were dried overnight at 45 °C in a drying oven (HK-D0100F, Hankuk General Equipment Plant, Hwaseong-si, Korea). The moisture contents of wheat grains germinated for 24, 48, 72, and 96 h were 8.6, 10.9, 12.4, and 12.8%, respectively. The dried germinated wheat samples were then milled into powder using a CyclotecTM 1093 sample mill (Foss, Hillerod, Denmark) and stored at −20 °C for further analysis. A wheat sample that was not soaked in water or germinated was used as the control in this study.

#### 3.1.2. Chemicals and Reagents

Acrylamide, acetonitrile, α-cyano-4-hydroxycinnamic acid, bis-acrylamide, benzamidine, Bradford solution, *p*-coumaric acid, caffeic acid, 3-[(3-cholamidopropyl)dimethylammonio]-1-propanesulfonate hydrate (CHAPS), 1,4-dithiothreitol (DTT), ferulic acid, fluorescein (FL), γ-aminobutyric acid (GABA), gallic acid, 4-hydroxybenzoic acid, iodoacetamide, phenylisothiocyanate, syringic acid, sodium dodecyl sulfate (SDS), Trolox, trifluoroacetic acid, thiourea, urea, and vanillic acid were purchased from Sigma Aldrich (St. Louis, MO, USA). Acetic acid, dipotassium phosphate, ethanol, hydrochloric acid, hexane, sodium hydroxide, monopotassium phosphate, methanol, and water were obtained from Junsei Chemical (Tokyo, Japan), and 2,2′-azobis(2-amidinoprpane) dihydrochloride solution (ABAP) was purchased from Wako Chemicals (Richmond, VA, USA). Pharmalyte (pH 3.5–10) and IPG Dry Strips (pH4–10 NL, 24 cm) were purchased from Amersham Biosciences and from Genomine Inc. (Pohang-si, Korea), respectively. Porcine trypsin was obtained from Promega (Madison, WI, USA). 

### 3.2. Methods

#### 3.2.1. Extraction of Free and Bound Phenolic Compounds

The extraction procedures for wheat phenolics were carried out according to a previous study [29]. For free phenolics, ground wheat grains were extracted with 80% chilled ethanol and the supernatants obtained by centrifugation (2500× *g* for 10 min) were evaporated to dryness at 45 °C using a nitrogen evaporator (Eyela MG-2200, Tokyo Rikakikai Co. Ltd., Tokyo, Japan). The dried material was then redissolved in methanol/hydrochloric acid (80:20, *v*/*v*) in a final volume of 5 mL. After the extraction of free phenolics, the residues were hydrolyzed with 6 M sodium hydroxide at room temperature for 1 h and then neutralized with hydrochloric acid to extract bound phenolics. The mixture was washed with hexane and extracted with ethyl acetate. The ethyl acetate fraction was evaporated to dryness at 45 °C, and redissolved in methanol/hydrochloric acid (80:20, *v*/*v*) in a final volume of 10 mL. The free and bound phenolics were used to determine the oxygen radical absorbance capacity (ORAC) and to identify individual phenolic acids.

#### 3.2.2. Oxygen Radical Absorbance Capacity (ORAC)

The ORAC values of the phenolic extracts were determined using a previously described method [6]. Briefly, 20 μL of diluted phenolic extract, phosphate buffer (blank), or Trolox (standard) and 200 μL of fluorescein disodium solution were transferred to black-walled 96-well plates. After incubator for 30 min at 37 °C, 20 μL of 2,2′-azobis(2-amidinopropane) dihydrochloride solution was added and the fluorescence intensity was measured every 60 s for 70 min using a SpectraMax^®^ i3 plate reader (Molecular Devices, Sunnyvale, CA, USA) at an excitation wavelength of 485 nm and an emission wavelength of 520 nm. The area under the curve (AUC) was calculated using Equation (1), where *f*1 = the fluorescence reading at cycle 1, *fi* = the fluorescence reading at cycle *i*, and CT = the cycle time in seconds.

(1) $$\mathrm{AUC}=\left[0.5+\overset{i=70}{\underset{i=1}{\sum }}fi/f1\right]\times CT$$

The ORAC value of each extract was calculated by comparison to the AUCs of a standard curve of Trolox. The ORAC values were expressed as micromoles of Trolox equivalents (TE) per gram of wheat sample expressed on a dry weight basis (µmol TE/g dwb).

#### 3.2.3. Phenolic Acid Compositions

The individual phenolic acids in germinated wheat extracts (free and bound) were separated by high-performance liquid chromatography (HPLC) on a system consisting of a Waters e2695 separation module (Waters Corporation, Milford, MA, USA) with a pump, an autoinjector, and a diode array detector (Waters 2998 photodiode array detector, Waters Corporation, Milford, MA, USA). The column and solvents used for chromatography and the flow rate and gradient programs were as previously described [30]. The resulting peaks were monitored at 280 nm and identified using the retention time and absorbance spectrum of a known standard for each compound. The phenolic acid contents were quantified by comparison to external calibration curves of each standard (gallic acid, 4-hydroxbenzoic acid, *p*-coumaric acid, vanillic acid, syringic acid, caffeic acid, and ferulic acid).

#### 3.2.4. Gamma-Aminobutyric Acid (GABA) Analysis

A known standard and 100 µL of each extract were converted to phenylthiocarbamyl (PTC) amino acid derivatives with phenylisothiocyanate (PITC). The PITC labeled samples were separated on a Pico-Tag column (Waters, 3.9 × 300 mm, 4 µm) by HPLC on a Water 510 system (Waters Corporation, Milford, MA) consisting of a pump, an autoinjector, and a detector (Waters 2487 UV detector, Waters Corporation, Milford, MA, USA). The mobile phase consisted of 140 mM sodium acetate containing 6% acetonitrile (solvent A) or 60% acetonitrile (solvent B). The total run time was 25 min at a constant flow rate of 1 mL/min with the following gradients, 100% A to 86% in 9 min; 86% A to 80% in 0.2 min; 80% A to 54% in 8.3 min; 54% A to 0% in 0.2 min; and 0% A to 100% in 7.3 min. The peaks of each component were monitored at 254 nm, and the peak acquired was processed using Empower software (Waters Corporation, Milford, MA, USA). The identification of each peak was confirmed using the retention time and absorbance spectrum of a known GABA standard. The GABA contents of each extract were quantified using external calibration curves of the GABA standard.

#### 3.2.5. Two-Dimensional gel Electrophoresis (2-DE)

About 0.5 g of germinated wheat grains were mixed with 1 mL of extraction buffer consisting of 7M urea, 2M thiourea containing 4% CHAPS, 1% DTT, 2% Pharmalyte, and 1mM benzamidine. Proteins were extracted for one h at room temperature with vortexing. After centrifugation at 12,000× *g* rpm for one hour at 25 °C, the supernatant was used for 2-DE. Protein concentrations were determined with the Bradford method [31].

Each protein sample extracted from germinated wheat samples (each about 200 µg) was loaded onto IPG strips, which were actively rehydrated at 30 V for 12 h at 20 °C in a Multiphor II apparatus (GE Healthcare, Little Chalfont, UK). For isoelectric focusing (IEF), the voltage was increased linearly from 150 to 3500 V over 3 h for sample entry, followed by a constant 3500 V with focusing complete after 96 kVh. Prior to the second dimension, the strips were incubated for 10 min in an equilibration buffer (50 mM Tris-Cl, pH 6.8 containing 6 M urea, 2% SDS, and 30% glycerol), first with 1% DTT and then with 2.5% iodoacetamide. The equilibrated strips were inserted onto SDS-PAGE gels (20 × 24 cm, 10–16%) and SDS-PAGE was performed using Hoefer DALT 2D system (Amersham Biosciences, Little Chalfont, UK) according to the manufacturer’s instructions. The 2D gels were run at 20 °C for 1700 Vh and were then silver stained as described previously [32], but the fixing and sensitization step with glutaraldehyde was omitted. The digitized images were quantitatively analyzed using the PDQuest software (version 7.0, BioRad, Hercules, CA, USA) according to the protocols provided by the manufacturer. The quantity of each spot was normalized to a total valid spot intensity. Protein spots were selected based on a significant expression level that deviated over 1.5 fold compared to the control or normal sample.

#### 3.2.6. Peptide Mass Fingerprinting (PMF)

For protein identification by PMF, protein spots were excised from the gel, digested with trypsin, mixed with α-cyano-4-hydroxycinnamic acid in 50% acetonitrile/0.1% TFA, and subjected to matrix-assisted laser desorption ionization-time of flight mass spectrometry (Microflex LRF 20, Bruker Daltonics, Billerica, MA, USA) as described previously [33]. Spectra were collected from 300 shots per spectrum over a range of 600 to 3000 *m*/*z* and calibrated by two-point internal calibration using trypsin auto-digestion peaks (*m*/*z* 842.5099, 2211.1046). A peak list was generated using Flex Analysis 3.0 (Bruker Daltonics, Billerica, MA, USA). The following thresholds were used for peak-picking: 500 for minimum resolution of monoisotopic mass, 5 for signal to noise (S/N). The search program MASCOT, developed by Matrixscience (http://www.matrixscience.com/), was used for protein identification by PMF. The following parameters were used for the database search: trypsin as the cleaving enzyme, a maximum of one missed cleavage, carbamidomethyl as fixed modifications, oxidation as variable modifications, monoisotopic masses and a mass tolerance of ±0.1 Da. The PMF acceptance criterion was the probability scoring.

#### 3.2.7. Statistical Analysis

Three replications of experiments were performed, except for 2-DE analysis, which was conducted in duplicate, and all data were presented as the mean ± standard deviation. An analysis of variance (ANOVA) was done to determine the differences among samples with regard to each characteristic tested in this study, and a Student Newman–Keuls (SNK) multiple comparison was performed to separate the means using Statistical Analysis Software (SAS, version 9.0, SAS Institute Inc., Cary, NC, USA). The correlation loading plots were obtained from a partial least square (PLS) regression analysis using XLSTAT (Version 2016; Addinosft, New York, NY, USA). A PLS regression analysis was conducted with the mean values of intensity of protein pattern spots by 2-DEs and beneficial compounds (as X variables), and the ORAC value (as Y variable) was used to understand the relationship between compounds related to protein pattern modification or phenolic compounds, as well as antioxidant properties in germinated wheat samples. 

## 4. Conclusions

Germinated cereal grains might be attractive food products or ingredients for health-conscious consumers. The germination process is an effective way to improve health-related parameters and the nutritional value of cereals. This study is the first to report on the relationships between changing protein patterns and antioxidant properties of wheat during germination. The present study indicated that a longer germination period resulted in higher levels of phenolic acids (gallic acid, 4-hydroxybenzoic acid, vanillic acid, caffeic acid, syringic acid, ferulic acid, and *p*-coumaric acid) and GABA than were found in ungerminated control wheat grains. The ORAC value, representing the antioxidant capacity, also gradually increased with the germination time. Overall, the antioxidant capacity of wheat samples with the longest (96 h) germination time exhibited a close relationship with phenolic acids, GABA, and proteins, such as glutathione *S*-transferase, granule bound starch synthase, and β-glucosidase. The results of the current study contribute to the knowledge of wheat protein pattern modifications during germination and their relationship to antioxidant properties. Further proteome analysis studies are needed to identify differences in the genetic backgrounds of germinated wheat grains in relation to their antioxidant properties.

## Figures and Tables

**Table 1:** Phenolic acid compositions and γ-aminobutyric acid (GABA) contents in ungerminated control wheat grains and in wheat grains at various times (24, 48, 72, and 96 h) during germination.

Phenolic Acids		Germination Time (h)				
		Control	24	48	72	96
Gallic acid (mg/100 g dwb ^1^)	Free ***	0.18 ± 0.03 ^c^	0.25 ± 0.07 ^c^	0.31 ± 0.07 ^bc^	0.60 ± 0.23 ^b^	1.57 ± 0.29 ^a^
	Bound ***	0.81 ± 0.09 ^c^	1.12 ± 0.12 ^b^	1.32 ± 0.09 ^b^	1.98 ± 0.20 ^a^	1.78 ± 0.21 ^a^
	Total ^2,^***	0.99 ± 007 ^d^	1.36 ± 0.20 ^cd^	1.63 ± 0.02 ^c^	2.58 ± 0.35 ^b^	3.36 ± 0.35 ^a^
4-Hydroxybenzoic acid (mg/100 g dwb ^1^)	Free ***	0.19 ± 0.07 ^c^	0.38 ± 0.08 ^c^	0.54 ± 0.12 ^bc^	0.91 ± 0.36 ^b^	4.70 ± 0.36 ^a^
	Bound ***	0.29 ± 0.03 ^c^	0.27 ± 0.07 ^c^	0.63 ± 0.08 ^b^	0.77 ± 0.11 ^ab^	0.85 ± 0.09 ^a^
	Total ^2,^***	0.47 ± 0.04 ^d^	0.65 ± 0.15 ^cd^	1.17 ± 0.04 ^bc^	1.68 ± 0.45 ^b^	5.55 ± 0.45 ^a^
Vanillic acid (mg/100 g dwb ^1^)	Free ***	0.16 ± 0.02 ^d^	0.32 ± 0.05 ^d^	0.77 ± 0.08 ^c^	2.79 ± 0.23 ^b^	3.72 ± 0.15 ^a^
	Bound ***	1.51 ± 0.12 ^c^	1.71 ± 0.16 ^c^	1.64 ± 0.17 ^c^	16.27 ± 0.31 ^b^	19.43 ± 3.09 ^a^
	Total ^2,^***	1.67 ± 0.13 ^c^	2.03 ± 0.18 ^c^	2.41 ± 0.25 ^c^	19.05 ± 0.26 ^b^	23.15 ± 3.24 ^a^
Caffeic acid (mg/100 g dwb ^1^)	Free ***	0.82 ± 0.13 ^d^	14.20 ± 0.33 ^c^	13.84 ± 0.61 ^c^	15.85 ± 0.78 ^b^	17.61 ± 0.30 ^a^
	Bound ***	0.11 ± 0.03 ^b^	0.09 ± 0.04 ^b^	0.10 ± 0.03 ^b^	0.07 ± 0.05 ^b^	0.38 ± 0.05 ^a^
	Total ^2,^***	0.93 ± 0.11 ^d^	14.29 ± 0.35 ^c^	13.94 ± 0.58 ^c^	15.92 ± 0.77 ^b^	17.99 ± 0.29 ^a^
Syringic acid (mg/100 g dwb ^1^)	Free ***	0.11 ± 0.07 ^b^	0.17 ± 0.04 ^b^	0.30 ± 0.09 ^b^	0.29 ± 0.06 ^b^	1.56 ± 0.20 ^a^
	Bound ***	0.34 ± 0.06 ^c^	0.39 ± 0.03 ^c^	0.38 ± 0.02 ^c^	0.51 ± 0.03 ^b^	0.67 ± 0.07 ^a^
	Total^2,^***	0.45 ± 0.13 ^c^	0.57 ± 0.05 ^c^	0.68 ± 0.10 ^bc^	0.80 ± 0.09 ^b^	2.22 ± 0.19 ^a^
Ferulic acid (mg/100 g dwb ^1^)	Free ***	1.15 ± 0.17 ^c^	1.04 ± 0.11 ^c^	1.13 ± 0.08 ^c^	1.62 ± 0.13 ^b^	1.51 ± 0.09 ^a^
	Bound ***	40.19 ± 1.70 ^d^	49.76 ± 4.46 ^c^	50.57 ± 1.22 ^b^	61.01 ± 2.56 ^b^	63.13 ± 0.77 ^a^
	Total ^2,^***	41.35 ± 1.60 ^e^	50.81 ± 4.39 ^d^	51.69 ± 1.14 ^c^	62.63 ± 2.54 ^b^	64.64 ± 0.69 ^a^
*p*-Coumaric acid (mg/100 g dwb ^1^)	Free ***	0.49 ± 0.06 ^b^	0.45 ± 0.06 ^b^	0.48 ± 0.04 ^b^	0.65 ± 0.05 ^a^	0.79 ± 0.09 ^a^
	Bound ***	1.48 ± 0.05 ^c^	2.61 ± 0.21 ^b^	3.57 ± 0.26 ^b^	3.87 ± 0.17 ^a^	4.22 ± 0.09 ^a^
	Total ^2,^***	1.97 ± 0.10 ^c^	3.06 ± 0.17 ^b^	4.06 ± 0.28 ^b^	4.52 ± 0.15 ^a^	5.01 ± 0.08 ^a^
GABA (mg/100 g dwb ^1^)	4.55 ± 0.28 ^a^	12.93 ± 0.48 ^b^	7.82 ± 0.06 ^b^	4.51 ± 0.26 ^d^	39.98 ± 1.77 ^a^

Data are means of three replications ± standard deviation. The same letter within a row indicates no significant difference. *** Significantly different at *p* < 0.001. ^1^ dwb means dry weight basis. ^2^ Total is sum of free and bound forms of each phenolic acid.

**Table 2:** Proteins detected on two-dimensional electrophoresis gels of ungerminated wheat grains and wheat grain samples germinated for various times (24, 48, 72, and 96 h).

Spot	Identified Proteins	Accession	Score ^2^	Mw	PI	Relative Expression of Proteins				
						Control	24 h	48 h	72 h	96 h
3104	Cytosolic malate dehydrogenase	AAP7009.1	98	22	6.0	3313	3459	2553	2355	1601
4011	Dimeric alpha-amylase inhibitor	ABI54569.1	88	14	6.6	215	176	87	77	ND
4101	Hypothetical protein CL3131Contig 1	AFG48690.1	69	24	6.3	339	204	188	191	ND
4010	Alpha-amylase inhibitor	CAI84642.1	96	14	6.6	279	202	183	187	ND
4008	Alpha-amylase/trypsin inhibitor	AQT26482.1	94	12	6.6	322	213	265	214	ND
5401	Phospholipase SGR2isoform	XP_022027330.1	86	38	6.8	505	499	361	430	262
1603	NI ^1^	-		56	5.3	139	153	122	85	85
2813	NI ^1^	-		88	5.6	753	1009	1926	1854	1854
3708	NI ^1^	-		63	5.7	191	192	99	142	537
3405	Granule bound starch synthase	AF75531.1	399	41	6.0	2189	2363	2362	2362	12,768
8009	Dimeric alpha-amylase inhibitor	AAV39517.1	153	11	8.2	380	1823	1367	1780	2599
9004	Glutathione S-transferase	XP_02255327.1	77	11	9.9	2581	5723	6173	6058	9776
4201	unnamed protein product	CDJ26374.1	91	27	6.4	414	672	794	586	954
8013	NI ^1^	-		11	8.9	ND	1939	2940	1884	3846
1801	Beta-glucosidase	XP_010066854.1	63	102	5.0	585	895	782	626	442

^1^ NI means not identified by Peptide Mass Fingerprinting. ^2^ Score indicates the Mowse score from mascot search results, and all scores are significant (*p* < 0.05). Values of relative expressions of proteins were means of two replications.

## Supplementary Materials

The following are available online. Figure S1: Images of proteins separated by two dimensional gel electrophoresis of wheat samples germinated for various periods (24, 48, 72, and 96 h) and ungerminated control, Figure S2: Heatmap showing the relative intensity of proteins (SSP) selected from two dimensional electrophoresis gels of wheat samples germinated for various periods (24, 48, 72, and 96 h) or ungerminated controls. (Regions I, II, III, and IV represent (I) high molecular weight-glutenin subunit, (II) ω-gliadins, (III) α/β and γ gliadins and low molecular weight glutenin subunit; (IV) nonstorage proteins.).
