# Supplementary material for: Effects of Germination on Protein, γ-Aminobutyric Acid, Phenolic Acids, and Antioxidant Capacity in Wheat
Source: Molecules. 2018 Sep 3;23(9):2244. doi: 10.3390/molecules23092244 (PMC6225431; doi:10.3390/molecules23092244)
Supplement: Supplementary file 1 [file molecules-23-02244-s001.zip › molecules-331387-supplementary/molecules-331387-supplementary.pdf]

Figure captions

Supplementary figure 1. Images of protein spots produced by two dimensional gel electrophoresis of wheat samples germinated for various periods (24, 48, 72, and 96 h) and control.

Supplementary figure 2. Heated map represented with relative intensity of selected spot proteins (SSP) from two dimensional gel electrophoresis of wheat samples germinated for various periods (24, 48, 72, and 96 h) in comparison to that of control. (Ⅰ, Ⅱ, Ⅲ, and Ⅳ regions represent (Ⅰ) high molecular weight-glutenin subunit, (Ⅱ) ω-gliadins, (Ⅲ) α/ß and γ gliadins and low molecular weight glutenin subunit; (Ⅳ) non-storage proteins, respectively.)

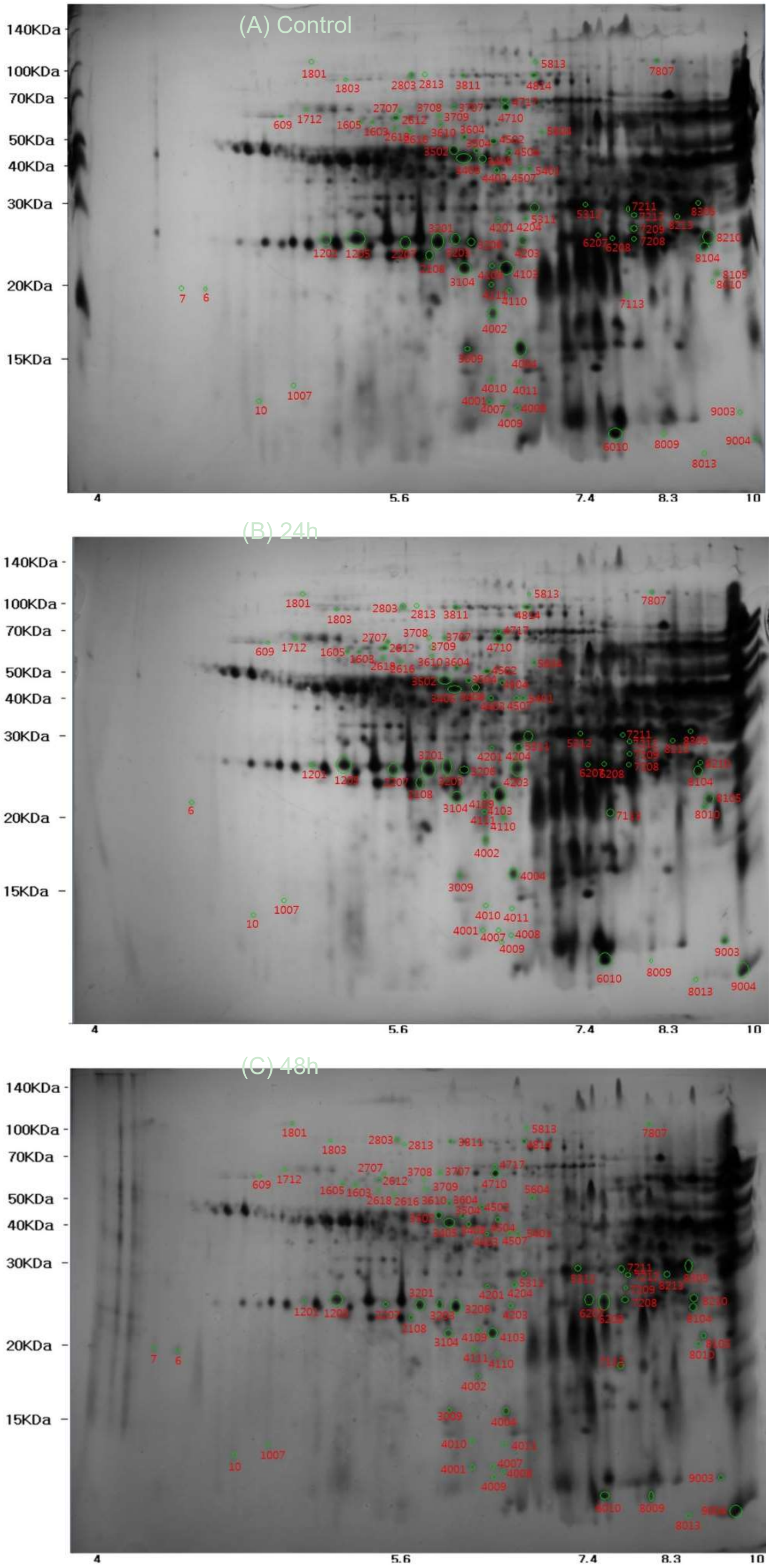
(A) Control
140KDa
100KDa
70KDa
50KDa
40KDa
30KDa
20KDa
15KDa
4
5.6
7.4
8.3
10
(B) 24h
(C) 48h

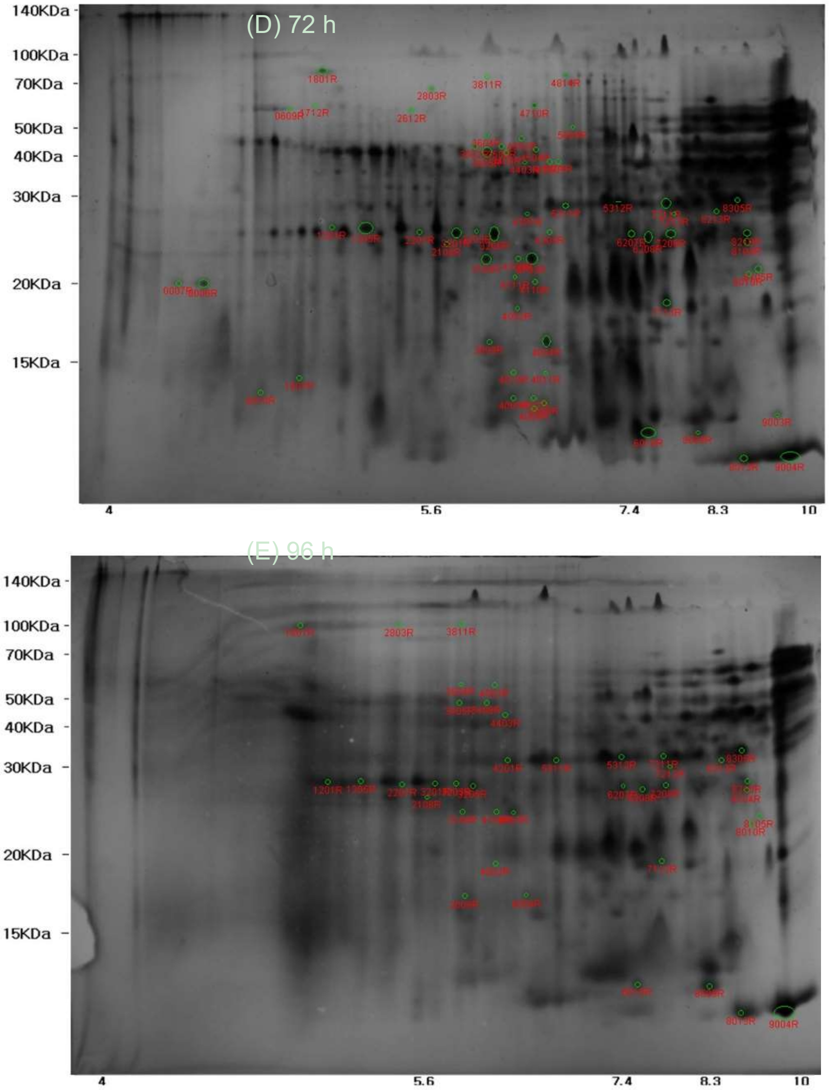

Supplementary figure 1

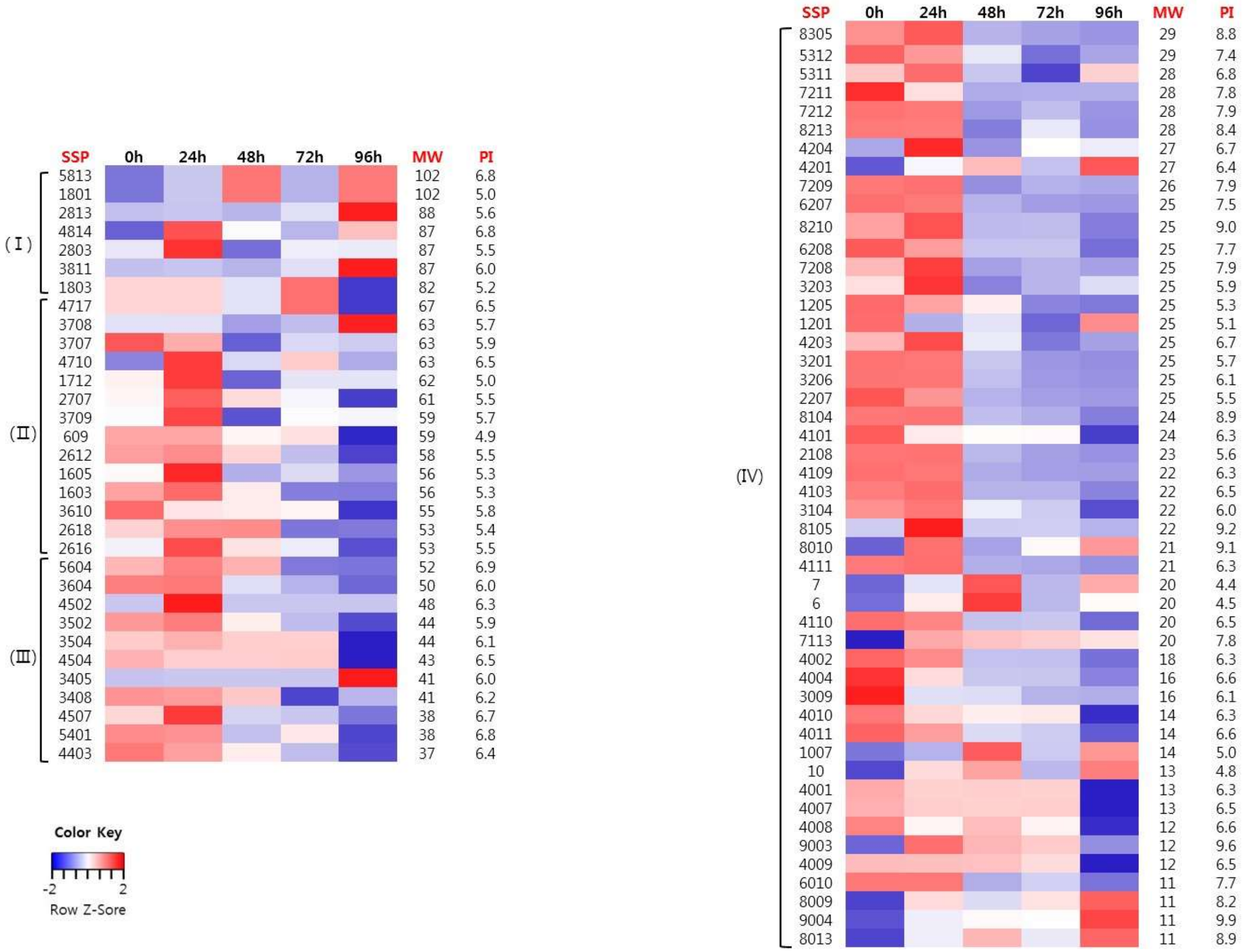

Supplementary figure 2
