# Supplementary material for: Effects of Germination on Protein, γ-Aminobutyric Acid, Phenolic Acids, and Antioxidant Capacity in Wheat
Source: Molecules. 2018 Sep 3;23(9):2244. doi: 10.3390/molecules23092244 (PMC6225431; doi:10.3390/molecules23092244)
Supplement: Supplementary file 1 [file molecules-23-02244-s001.zip › molecules-331387-supplementary/Peptide list/peptide list_1801.pdf]

MATRIX SCIENCE MASCOT Search Results

Protein View: XP_010066854.1

PREDICTED: beta-glucosidase 40 [Eucalyptus grandis]

**Database:**	NCBIprot
**Score:**	63
**Expect:**	2.7
**Monoisotopic mass (M_r):**	57772
**Calculated pI:**	6.36
**Taxonomy:**	Eucalyptus grandis

This protein sequence matches the following other entries:

- KCW64896.1 from Eucalyptus grandis

Sequence similarity is available as an NCBI BLAST search of XP_010066854.1 against nr.

Search parameters

**Enzyme:**	Trypsin: cuts C-term side of KR unless next residue is P.
**Fixed modifications:**	Carbamidomethyl (C)
**Variable modifications:**	Oxidation (M)
**Mass values searched:**	23
**Mass values matched:**	7

Protein sequence coverage: 21%

Matched peptides shown in ***bold red***.

1 MERYRPR**AAA ASAMMAGLLA GLLVHACCAD AIGR**SSFPKG FVFGTASSAF
51 QYEGAVKEDG RGPSVWDTFS HQFGKVIDLS NADVAVDQYH RFNEDIQLMK
101 DMGMDAYRFS ISWSRIFPNG SGQINQAGVD HYNNLINALL AKGIEPYVTL
151 FHWDLPQALE DKYNGWLDPQ IIKDFATYAE TCFQKFGDRV KHWVTFNEPH
201 TLAIQGYDVG LQAPGR**CSIL LHLFCR**AGNS ATEPYIVGHN ILLSHATAVD
251 VYRKKYKQKQ HGSIGVAFDV MWFVPR**TNST DDIEATQR**AL DFQFGWFIEP
301 LIFGNYPSSM ISRVGSRLPA ISSSESALLK GSLDFVGINH YTTYYGSNDT
351 SDVIGSLLKD SLSDSGSVTL PFRDGILNPI GDRASSIWLY IVPQGLR**SLM**
401 **NYIKTK**YGNP PVIITENGMD DPNPPLINIK EALKDEKR**IK YHNDYLTNLL**
451 **ASIKEDGCNV KGYFVWSLLD NWEWAAGYTS R**FGLYFVDYN DKLKRYPK**DS**
501 **VQWFK**NFLKS T

Unformatted sequence string: 511 residues (for pasting into other applications).

Sort by ◉ residue number ○ increasing mass ○ decreasing mass
Show ◉ matched peptides only ○ predicted peptides also

Start – End	Observed	Mr(expt)	Mr(calc)	Delta	M	Peptide
8 – 34	2658.2607	2657.2535	2657.2910	-0.0375	0	R.AAAASAMMAGLLAGLLVHACCADAIGR.S + Oxidation (M)
217 – 226	1318.6462	1317.6389	1317.6686	-0.0297	0	R.CSILLHLFCR.A
277 – 288	1350.6440	1349.6367	1349.6059	0.0308	0	R.TNSTDDIEATQR.A
398 – 406	1097.4415	1096.4342	1096.5950	-0.1608	1	R.SLMNYIKTK.Y
439 – 454	1905.9754	1904.9681	1905.0359	-0.0679	1	R.IKYHNDYLTNLLASIK.E
455 – 481	3223.3126	3222.3054	3222.4505	-0.1452	1	K.EDGCNVKGYFVWSLLDNWEWAAGYTSR.F
499 – 505	909.3471	908.3398	908.4392	-0.0994	0	K.DSVQWFK.N

No match to: 842.3830, 856.3992, 870.4262, 948.3295, 1125.4659, 1187.6122, 1232.5516, 1326.6202, 1339.5863, 1372.6291, 1650.8046, 1766.7704, 1794.7974, 2055.9753, 2211.1043, 3621.4175

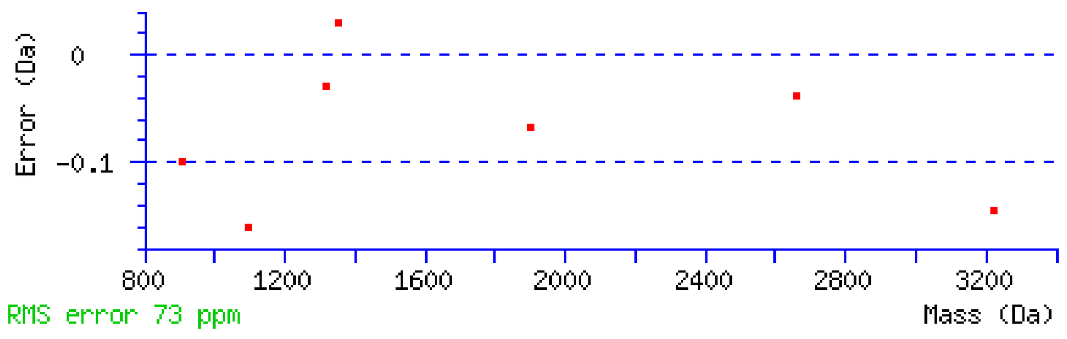

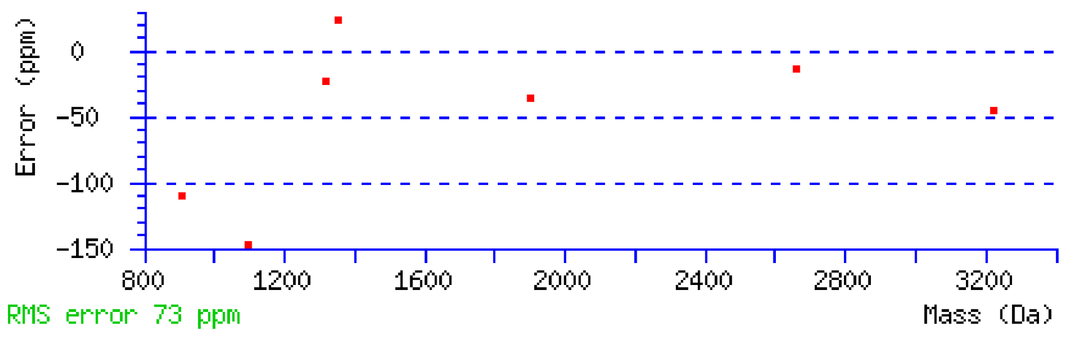


```
LOCUS       XP_010066854             511 aa            linear   PLN 25-OCT-2016
DEFINITION  PREDICTED: beta-glucosidase 40 [Eucalyptus grandis].
ACCESSION   XP_010066854
VERSION     XP_010066854.1
DBLINK      BioProject: PRJNA264012
DBSOURCE    REFSEQ: accession XM_010068552.2
KEYWORDS    RefSeq.
SOURCE      Eucalyptus grandis
  ORGANISM  Eucalyptus grandis
            Eukaryota; Viridiplantae; Streptophyta; Embryophyta; Tracheophyta;
            Spermatophyta; Magnoliophyta; eudicotyledons; Gunneridae;
            Pentapetalae; rosids; malvids; Myrtales; Myrtaceae; Myrtoideae;
            Eucalypteae; Eucalyptus.
COMMENT     MODEL REFSEQ:  This record is predicted by automated computational
            analysis. This record is derived from a genomic sequence
            (NW_010092444.1) annotated using gene prediction method: Gnomon,
            supported by EST evidence.
            Also see:
                Documentation of NCBI's Annotation Process

            ##Genome-Annotation-Data-START##
            Annotation Provider         :: NCBI
            Annotation Status           :: Full annotation
            Annotation Version          :: Eucalyptus grandis Annotation
                                           Release 101
            Annotation Pipeline         :: NCBI eukaryotic genome annotation
                                           pipeline
            Annotation Software Version :: 7.2
            Annotation Method           :: Best-placed RefSeq; Gnomon
            Features Annotated          :: Gene; mRNA; CDS; ncRNA
            ##Genome-Annotation-Data-END##
            COMPLETENESS: full length.
FEATURES             Location/Qualifiers
     source          1..511
                     /organism="Eucalyptus grandis"
                     /cultivar="BRASUZ1"
                     /db_xref="taxon:71139"
                     /chromosome="Unknown"
                     /tissue_type="leaf extractions"
                     /collection_date="Dec-2008"
                     /country="Brazil"
                     /note="individual tree from a line of selfed trees grown
                     from seeds collected in Coffs Harbor, Australia"
     Protein         1..511
                     /product="beta-glucosidase 40"
                     /calculated_mol_wt=57335
     Region          34..510
                     /region_name="Glyco_hydro_1"
                     /note="Glycosyl hydrolase family 1; cl23725"
                     /db_xref="CDD:304882"
     CDS             1..511
                     /gene="LOC104453907"
                     /coded_by="XM_010068552.2:286..1821"
                     /db_xref="GeneID:104453907"
```
