# Supplementary material for: Effects of Germination on Protein, γ-Aminobutyric Acid, Phenolic Acids, and Antioxidant Capacity in Wheat
Source: Molecules. 2018 Sep 3;23(9):2244. doi: 10.3390/molecules23092244 (PMC6225431; doi:10.3390/molecules23092244)
Supplement: Supplementary file 1 [file molecules-23-02244-s001.zip › molecules-331387-supplementary/Peptide list/peptide list_3104.pdf]

MASCOT Search Results

Protein View: AAP70009.1

cytosolic malate dehydrogenase, partial [Triticum aestivum]

**Database:**	NCBIprot
**Score:**	98
**Expect:**	0.00096
**Monoisotopic mass (M_r):**	24560
**Calculated pI:**	6.60
**Taxonomy:**	Triticum aestivum

Sequence similarity is available as an NCBI BLAST search of AAP70009.1 against nr.

Search parameters

**Enzyme:**	Trypsin: cuts C-term side of KR unless next residue is P.
**Fixed modifications:**	Carbamidomethyl (C)
**Variable modifications:**	Oxidation (M)
**Mass values searched:**	11
**Mass values matched:**	6

Protein sequence coverage: 41%

Matched peptides shown in ***bold red***.

```
  1 MLGADQPVIL HMLDIEFAAE ALKGVKMELI DAAFPLLKGV VATTDVVEAC
 51 TGVNVAVMVG GFPRKEGMER KDVMTKNVSI YKAQASALEA HAAPNCKVLV
101 VANPANTNAL ILKEFAPSIP EKNISCLTRL DHNRALGQIS ERLGVQVSDV
151 KNAIIWGNHS SSQYPDVNHA TVKTPSGEKP VRELVQDDEW LNGEFIATVQ
201 QRGAAIIKAR KLSSALSAAS SACDHIR
```

Matched (bold red) residues: 27–38 (MELI DAAFPLLK), 98–129 (VLV VANPANTNAL ILKEFAPSIP EKNISCLTR), 152–202 (NAIIWGNHS SSQYPDVNHA TVKTPSGEKP VRELVQDDEW LNGEFIATVQ QR).

Unformatted sequence string: 227 residues (for pasting into other applications).

Sort by ◉ residue number ○ increasing mass ○ decreasing mass
Show ◉ matched peptides only ○ predicted peptides also

Start – End	Observed	Mr(expt)	Mr(calc)	Delta	M	Peptide
27 – 38	1360.7031	1359.6958	1359.7472	-0.0514	0	K.MELIDAAFPLLK.G
98 – 113	1649.9419	1648.9346	1648.9876	-0.0530	0	K.VLVVANPANTNALILK.E
114 – 129	1861.9237	1860.9164	1860.9404	-0.0239	1	K.EFAPSIPEKNISCLTR.L
152 – 173	2438.1844	2437.1771	2437.1775	-0.0003	0	K.NAIIWGNHSSSQYPDVNHATVK.T
174 – 202	3341.8294	3340.8221	3340.6688	0.1533	1	K.TPSGEKPVRELVQDDEWLNGEFIATVQQR.G
183 – 202	2390.1531	2389.1458	2389.1550	-0.0092	0	R.ELVQDDEWLNGEFIATVQQR.G

No match to: 1350.6246, 1641.7904, 1905.9620, 2211.1040, 2373.1022

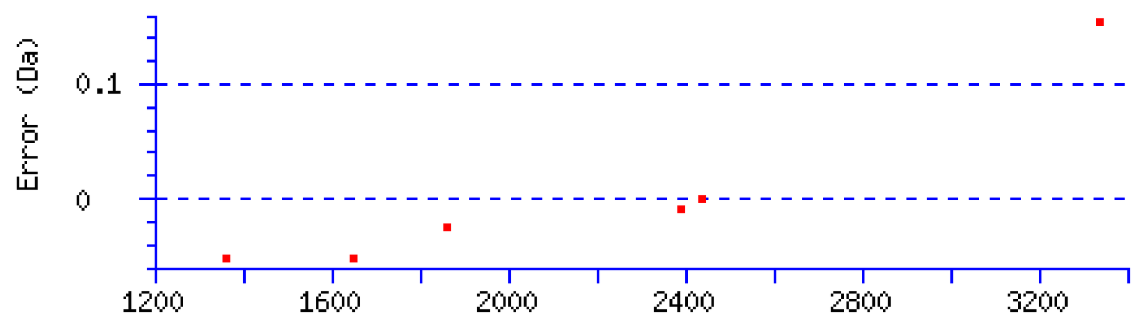

RMS error 28 ppm

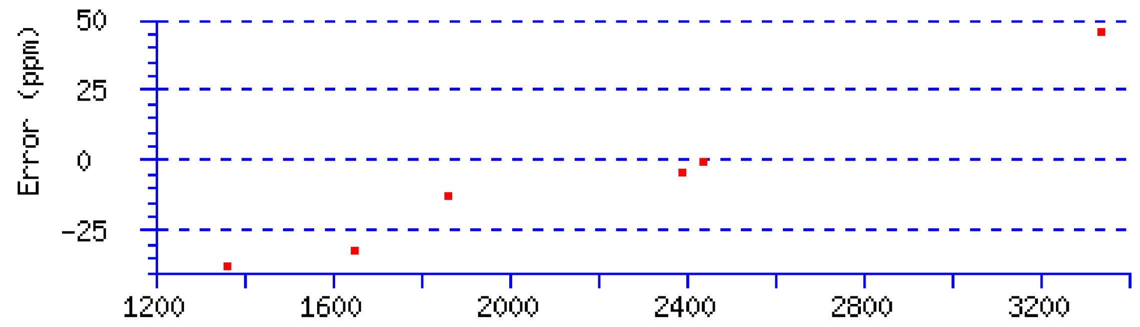

RMS error 28 ppm

```
LOCUS       AAP70009                 227 aa            linear   PLN 26-JUL-2016
DEFINITION  cytosolic malate dehydrogenase, partial [Triticum aestivum].
ACCESSION   AAP70009
VERSION     AAP70009.1
DBSOURCE    accession AY236152.1
KEYWORDS    .
SOURCE      Triticum aestivum (bread wheat)
  ORGANISM  Triticum aestivum
            Eukaryota; Viridiplantae; Streptophyta; Embryophyta; Tracheophyta;
            Spermatophyta; Magnoliophyta; Liliopsida; Poales; Poaceae; BOP
            clade; Pooideae; Triticodae; Triticeae; Triticinae; Triticum.
REFERENCE   1  (residues 1 to 227)
  AUTHORS   Ma,Q.-H.
  TITLE     Characterization of a cytosolic malate dehydrogenase cDNA from
            wheat
  JOURNAL   Unpublished
REFERENCE   2  (residues 1 to 227)
  AUTHORS   Ma,Q.-H. Sr.
  TITLE     Direct Submission
  JOURNAL   Submitted (13-FEB-2003) Key Laboratory of Photosynthesis and
            Environmental Molecular Physiology, Institute of Botany, Chinese
            Academy of Sciences, 20 Nanxin Cun, Xiangshan, Beijing 100093,
            China
COMMENT     Method: conceptual translation supplied by author.
FEATURES             Location/Qualifiers
     source          1..227
                     /organism="Triticum aestivum"
                     /cultivar="H4564"
                     /db_xref="taxon:4565"
     Protein         <1..>227
                     /product="cytosolic malate dehydrogenase"
                     /EC_number="1.1.1.37"
     Region          1..>227
                     /region_name="NADB_Rossmann"
                     /note="Rossmann-fold NAD(P)(+)-binding proteins; cl21454"
                     /db_xref="CDD:304358"
     Site            order(14..15,59..60,80,101..103,127,130,159,218)
                     /site_type="other"
                     /note="NAD(P) binding site [chemical binding]"
                     /db_xref="CDD:133419"
     Site            order(21,25,27..28,30..32,133..134,136..137,143,220,224)
                     /site_type="other"
                     /note="LDH/MDH dimer interface [polypeptide binding]"
                     /db_xref="CDD:133419"
     Site            order(103,134,159)
                     /site_type="other"
                     /note="substrate binding site [chemical binding]"
                     /db_xref="CDD:133419"
     CDS             1..227
                     /coded_by="AY236152.1:<1..>683"
```
