# Supplementary material for: Effects of Germination on Protein, γ-Aminobutyric Acid, Phenolic Acids, and Antioxidant Capacity in Wheat
Source: Molecules. 2018 Sep 3;23(9):2244. doi: 10.3390/molecules23092244 (PMC6225431; doi:10.3390/molecules23092244)
Supplement: Supplementary file 1 [file molecules-23-02244-s001.zip › molecules-331387-supplementary/Peptide list/peptide list_3405.pdf]

MATRIX SCIENCE MASCOT Search Results

Protein View: AFC75531.1

granule bound starch synthase, partial [Triticum spelta]

**Database:**	NCBIprot
**Score:**	399
**Expect:**	7.3e-34
**Monoisotopic mass (M_r):**	64252
**Calculated pI:**	8.61
**Taxonomy:**	Triticum spelta

Sequence similarity is available as an NCBI BLAST search of AFC75531.1 against nr.

Search parameters

**Enzyme:**	Trypsin: cuts C-term side of KR unless next residue is P.
**Fixed modifications:**	Carbamidomethyl (C)
**Variable modifications:**	Oxidation (M)
**Mass values searched:**	43
**Mass values matched:**	37

Protein sequence coverage: 55%

Matched peptides shown in ***bold red***.

```
  1 MAALVTSQLA TSGTVLGITD RFRRAGFQGV RPRSPADAAL GMRTVGASAA
 51 PTQSRKAHRG TRRCLSMVVR ATGSGGMNLV FVGAEMAPWS KTGGLGDVLG
101 GLPPAMAANG HRVMVISPRY DQYKDAWDTS VVSEIKVVDK YERVRYFHCY
151 KRGVDRVFVD HPCFLEKVRG KTKEKIYGPD AGTDYEDNQQ RFSLLCQAAL
201 EVPRILNLDN NPYFSGPYED VVFVCNDWHT GLLACYLKSN YQSNGIYRAA
251 KVAFCIHNIS YQGRFSFDDF AQLNLPDRFK SSFDFIDGYD KPVEGRKINW
301 MKAGILQADK VLTVSPYYAE ELISGEARGC ELDNIMRLTG ITGIVNGMDV
351 SEWDPTKDKF LAVNYDITTA LEGKALNKEA LQAEVGLPVD RKVPLVAFIG
401 RLEEQKGPDV MIAAIPEILK EEDVQIVLLG TGKKKFERLL KSIEEKFPSK
451 VRAVVRFNAP LAHQMMAGAD VLAVTSRFEP CGLIQLQGMR YGTPCACAST
501 GGLVDTIVEG KTGFHMGRLS VDCNVVEPAD VKKVVTTLKR AVKVVGTPAY
551 HEMVKNCMIQ DLSWKGPAKN WEDVLL
```

Unformatted sequence string: 576 residues (for pasting into other applications).

Sort by ◉ residue number ○ increasing mass ○ decreasing mass
Show ◉ matched peptides only ○ predicted peptides also

Start – End	Observed	Mr(expt)	Mr(calc)	Delta	M	Peptide
71 – 91	2109.9739	2108.9666	2109.0023	-0.0357	0	R.ATGSGGMNLVFVGAEMAPWSK.T
71 – 91	2125.9971	2124.9898	2124.9973	-0.0075	0	R.ATGSGGMNLVFVGAEMAPWSK.T + Oxidation (M)
92 – 112	1976.9737	1975.9664	1975.9898	-0.0234	0	K.TGGLGDVLGGLPPAMAANGHR.V + Oxidation (M)
113 – 119	801.3313	800.3240	800.4578	-0.1338	0	R.VMVISPR.Y
120 – 136	2046.9584	2045.9511	2045.9582	-0.0071	1	R.YDQYKDAWDTSVVSEIK.V
144 – 151	1172.5121	1171.5048	1171.5597	-0.0549	1	R.VRYFHCYK.R
146 – 151	917.2917	916.2844	916.3902	-0.1057	0	R.YFHCYK.R
146 – 152	1073.4323	1072.4250	1072.4913	-0.0663	1	R.YFHCYKR.G
153 – 167	1817.8849	1816.8776	1816.8931	-0.0154	1	R.GVDRVFVDHPCFLEK.V
157 – 167	1390.6532	1389.6459	1389.6751	-0.0292	0	R.VFVDHPCFLEK.V
157 – 169	1645.8496	1644.8423	1644.8447	-0.0023	1	R.VFVDHPCFLEKVR.G
174 – 191	2098.9956	2097.9884	2097.9239	0.0645	1	K.EKIYGPDAGTDYEDNQQR.F
176 – 191	1841.7834	1840.7761	1840.7864	-0.0103	0	K.IYGPDAGTDYEDNQQR.F
239 – 248	1201.5049	1200.4976	1200.5523	-0.0547	0	K.SNYQSNGIYR.A
239 – 251	1471.7928	1470.7856	1470.7215	0.0641	1	K.SNYQSNGIYRAAK.V
265 – 278	1684.7812	1683.7739	1683.7893	-0.0154	0	R.FSFDDFAQLNLPDR.F
265 – 280	1959.9585	1958.9513	1958.9527	-0.0014	1	R.FSFDDFAQLNLPDRFK.S
279 – 296	2106.9959	2105.9886	2106.0058	-0.0172	1	R.FKSSFDFIDGYDKPVEGR.K
281 – 296	1831.8337	1830.8264	1830.8425	-0.0161	0	K.SSFDFIDGYDKPVEGR.K
298 – 310	1503.7733	1502.7660	1502.7915	-0.0255	1	K.INWMKAGILQADK.V + Oxidation (M)
311 – 328	1997.0123	1996.0051	1996.0153	-0.0102	0	K.VLTVSPYYAEELISGEAR.G
329 – 337	1107.4274	1106.4202	1106.4848	-0.0647	0	R.GCELDNIMR.L
375 – 391	1822.9875	1821.9802	1821.9948	-0.0146	1	K.ALNKEALQAEVGLPVDR.K
379 – 391	1396.7055	1395.6982	1395.7358	-0.0376	0	K.EALQAEVGLPVDR.K
392 – 401	1099.6348	1098.6275	1098.6913	-0.0638	1	R.KVPLVAFIGR.L
393 – 401	971.5152	970.5079	970.5964	-0.0885	0	K.VPLVAFIGR.L
457 – 477	2200.0641	2199.0568	2199.0929	-0.0360	0	R.FNAPLAHQMMAGADVLAVTSR.F
457 – 477	2216.0657	2215.0584	2215.0878	-0.0293	0	R.FNAPLAHQMMAGADVLAVTSR.F + Oxidation (M)
478 – 490	1548.7453	1547.7381	1547.7588	-0.0208	0	R.FEPCGLIQLQGMR.Y
478 – 490	1564.7453	1563.7380	1563.7538	-0.0158	0	R.FEPCGLIQLQGMR.Y + Oxidation (M)
491 – 511	2156.0014	2154.9941	2154.9926	0.0015	0	R.YGTPCACASTGGLVDTIVEGK.T
512 – 518	805.2412	804.2339	804.3701	-0.1362	0	K.TGFHMGR.L
512 – 518	821.2493	820.2420	820.3650	-0.1230	0	K.TGFHMGR.L + Oxidation (M)
519 – 533	1672.8402	1671.8329	1671.8502	-0.0172	1	R.LSVDCNVVEPADVKK.V
534 – 540	816.3950	815.3877	815.5229	-0.1352	1	K.VVTTLKR.A
544 – 555	1330.6479	1329.6406	1329.6751	-0.0345	0	K.VVGTPAYHEMVK.N
544 – 555	1346.6577	1345.6505	1345.6700	-0.0196	0	K.VVGTPAYHEMVK.N + Oxidation (M)

No match to: 1319.6901, 1326.6402, 1412.7463, 1430.7624, 1446.7509, 2211.1040

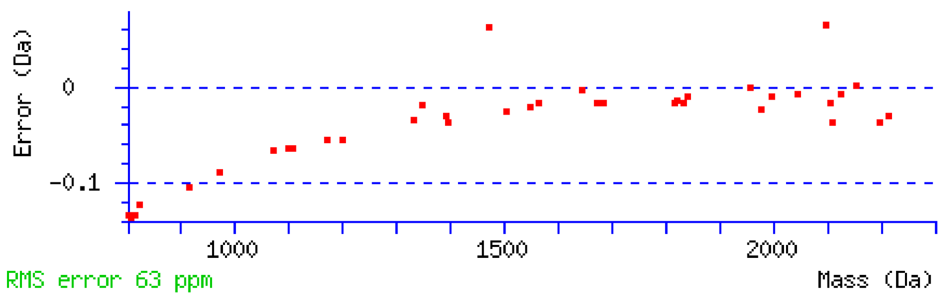

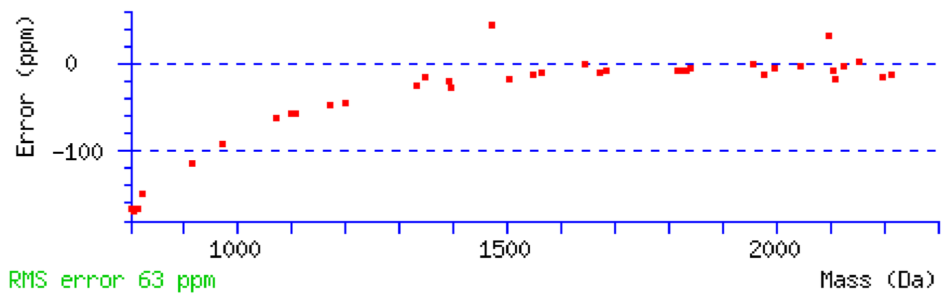


```
LOCUS       AFC75531                 576 aa            linear   PLN 06-MAR-2012
DEFINITION  granule bound starch synthase, partial [Triticum spelta].
ACCESSION   AFC75531
VERSION     AFC75531.1
DBSOURCE    accession JN935594.1
KEYWORDS    .
SOURCE      Triticum spelta
  ORGANISM  Triticum spelta
            Eukaryota; Viridiplantae; Streptophyta; Embryophyta; Tracheophyta;
            Spermatophyta; Magnoliophyta; Liliopsida; Poales; Poaceae; BOP
            clade; Pooideae; Triticodae; Triticeae; Triticinae; Triticum.
REFERENCE   1  (residues 1 to 576)
  AUTHORS   Guzman,C., Leonor,C., Martin,L.M. and Alvarez,J.B.
  TITLE     Comparative analysis of the waxy genes present in Spanish spelt
            wheat with their homologous genes in polyploid wheat and relatives
  JOURNAL   Unpublished
REFERENCE   2  (residues 1 to 576)
  AUTHORS   Guzman,C., Leonor,C., Martin,L.M. and Alvarez,J.B.
  TITLE     Direct Submission
  JOURNAL   Submitted (26-OCT-2011) Genetics, University of Cordoba, Edificio
            C5 Gregor Mendel, Campus de Rabanales, Cordoba 14071, Spain
FEATURES             Location/Qualifiers
     source          1..576
                     /organism="Triticum spelta"
                     /isolate="D CGN 11460"
                     /db_xref="taxon:58933"
     Protein         1..>576
                     /product="granule bound starch synthase"
     Region          77..575
                     /region_name="glgA"
                     /note="glycogen/starch synthase, ADP-glucose type;
                     TIGR02095"
                     /db_xref="CDD:273969"
     Region          78..575
                     /region_name="GT1_Glycogen_synthase_DULL1_like"
                     /note="This family is most closely related to the GT1
                     family of glycosyltransferases. Glycogen synthase
                     catalyzes the formation and elongation of the
                     alpha-1,4-glucose backbone using ADP-glucose, the second
                     and key step of glycogen biosynthesis. This family...;
                     cd03791"
                     /db_xref="CDD:99965"
     Site            order(91,399..401,456..457,462,479,484)
                     /site_type="other"
                     /note="ADP-binding pocket [chemical binding]"
                     /db_xref="CDD:99965"
     Site            order(320,499,504..505,507,536)
                     /site_type="other"
                     /note="homodimer interface [polypeptide binding]"
                     /db_xref="CDD:99965"
     CDS             1..576
                     /gene="Waxy"
                     /coded_by="join(JN935594.1:32..352,JN935594.1:443..523,
                     JN935594.1:619..717,JN935594.1:822..975,
                     JN935594.1:1131..1228,JN935594.1:1370..1723,
                     JN935594.1:1809..1988,JN935594.1:2071..2262,
                     JN935594.1:2347..2433,JN935594.1:2532..2660,
                     JN935594.1:2777..>2810)"
```

Mascot: http://www.matrixscience.com/
