# Supplementary material for: Effects of Germination on Protein, γ-Aminobutyric Acid, Phenolic Acids, and Antioxidant Capacity in Wheat
Source: Molecules. 2018 Sep 3;23(9):2244. doi: 10.3390/molecules23092244 (PMC6225431; doi:10.3390/molecules23092244)
Supplement: Supplementary file 1 [file molecules-23-02244-s001.zip › molecules-331387-supplementary/Peptide list/peptide list_4008.pdf]

MATRIX SCIENCE MASCOT Search Results

Protein View: AQT26482.1

alpha amylase/trypsin inhibitor, partial [Triticum aestivum]

**Database:**	NCBIprot
**Score:**	94
**Expect:**	0.0021
**Monoisotopic mass (M_r):**	15430
**Calculated pI:**	7.67
**Taxonomy:**	Triticum aestivum

Sequence similarity is available as an NCBI BLAST search of AQT26482.1 against nr.

Search parameters

**Enzyme:**	Trypsin: cuts C-term side of KR unless next residue is P.
**Fixed modifications:**	Carbamidomethyl (C)
**Variable modifications:**	Oxidation (M)
**Mass values searched:**	8
**Mass values matched:**	5

Protein sequence coverage: 63%

Matched peptides shown in ***bold red***.

1 RTNLLPHCR**D YVLQQTCGTF TPGSKLPEWM TSASIYSPGK PYLAK**LYCCQ
51 ELAEISQQCR CEALR**YFIAL PVPSQPVDPR SGNVGESGLI DLPGCPR**QMQ
101 WDFVR**LLVAP GQCNLATIHN VR**YCPAVEQP LWI

Unformatted sequence string: 133 residues (for pasting into other applications).

Sort by ◉ residue number ○ increasing mass ○ decreasing mass
Show ◉ matched peptides only ○ predicted peptides also

Start - End	Observed	Mr(expt)	Mr(calc)	Delta	M	Peptide
10 - 25	1801.7797	1800.7724	1800.8353	-0.0628	0	R.DYVLQQTCGTFTPGSK.L
26 - 45	2238.9806	2237.9733	2238.1394	-0.1661	0	K.LPEWMTSASIYSPGKPYLAK.L
66 - 80	1698.8806	1697.8733	1697.9141	-0.0408	0	R.YFIALPVPSQPVDPR.S
81 - 97	1727.8040	1726.7968	1726.8308	-0.0341	0	R.SGNVGESGLIDLPGCPR.Q
106 - 122	1875.9639	1874.9567	1875.0149	-0.0582	0	R.LLVAPGQCNLATIHNVR.Y

No match to: 1765.7308, 1794.7927, 2211.1040

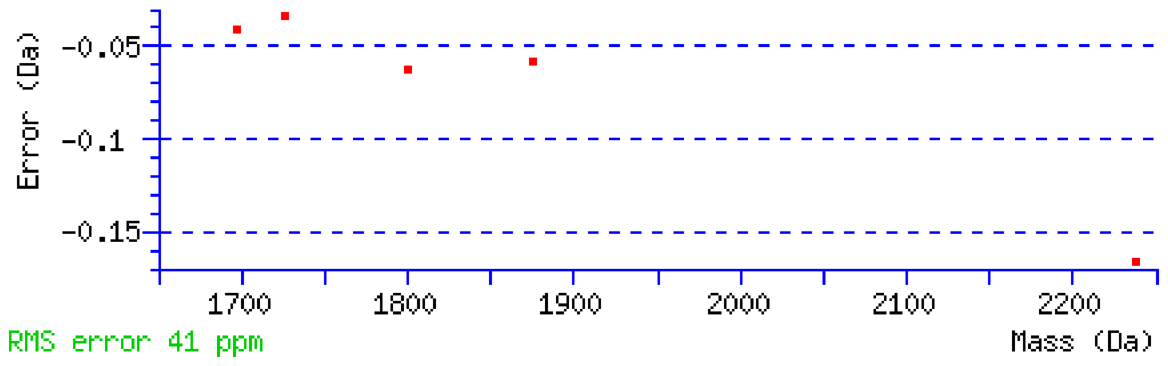

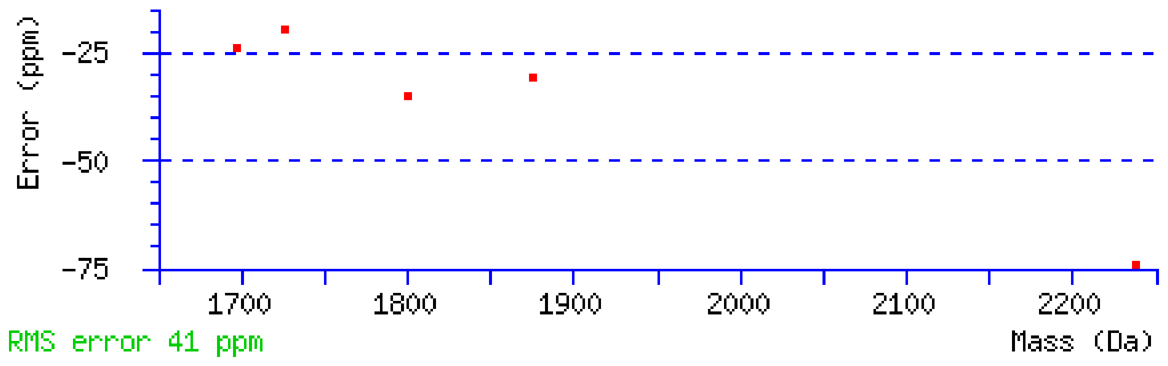


```
LOCUS       AQT26482                 133 aa            linear   PLN 04-MAR-2017
DEFINITION  alpha amylase/trypsin inhibitor, partial [Triticum aestivum].
ACCESSION   AQT26482
VERSION     AQT26482.1
DBSOURCE    accession KY274454.1
KEYWORDS    .
SOURCE      Triticum aestivum (bread wheat)
  ORGANISM  Triticum aestivum
            Eukaryota; Viridiplantae; Streptophyta; Embryophyta; Tracheophyta;
            Spermatophyta; Magnoliophyta; Liliopsida; Poales; Poaceae; BOP
            clade; Pooideae; Triticodae; Triticeae; Triticinae; Triticum.
REFERENCE   1  (residues 1 to 133)
  AUTHORS   Kaushik,M., Sinha,S.K. and Mandal,P.K.
  TITLE     Cloning and sequence analysis of alpha amylase/trypsin inhibitor
            gene from bread wheat (Triticum aestivum)
  JOURNAL   Unpublished
REFERENCE   2  (residues 1 to 133)
  AUTHORS   Kaushik,M., Sinha,S.K. and Mandal,P.K.
  TITLE     Direct Submission
  JOURNAL   Submitted (30-NOV-2016) NRCPB, ICAR-National Research Centre on
            Plant Biotechnology, Pusa Campus, New Delhi, Delhi 110012, India
FEATURES             Location/Qualifiers
     source          1..133
                     /organism="Triticum aestivum"
                     /cultivar="HD2967"
                     /db_xref="taxon:4565"
                     /tissue_type="seed"
                     /country="India"
     Protein         <1..>133
                     /product="alpha amylase/trypsin inhibitor"
     Region          5..125
                     /region_name="AAI_SS"
                     /note="Alpha-Amylase Inhibitors (AAIs) and Seed Storage
                     (SS) Protein subfamily; composed of cereal-type AAIs and
                     SS proteins. They are mainly present in the seeds of a
                     variety of plants. AAIs play an important role in the
                     natural defenses of plants against...; cd00261"
                     /db_xref="CDD:238163"
     Site            order(45,104,108,114,123)
                     /site_type="other"
                     /note="dimer interface [polypeptide binding]"
                     /db_xref="CDD:238163"
     CDS             1..133
                     /gene="ATI"
                     /coded_by="KY274454.1:<1..>403"
                     /codon_start=3
```
