# Supplementary material for: Effects of Germination on Protein, γ-Aminobutyric Acid, Phenolic Acids, and Antioxidant Capacity in Wheat
Source: Molecules. 2018 Sep 3;23(9):2244. doi: 10.3390/molecules23092244 (PMC6225431; doi:10.3390/molecules23092244)
Supplement: Supplementary file 1 [file molecules-23-02244-s001.zip › molecules-331387-supplementary/Peptide list/peptide list_4010.pdf]

MATRIX SCIENCE MASCOT Search Results

Protein View: CAI84642.1

alpha-amylase inhibitor 0.19, partial [Triticum aestivum]

**Database:**	NCBIprot
**Score:**	96
**Expect:**	0.0014
**Monoisotopic mass (M_r):**	13340
**Calculated pI:**	6.86
**Taxonomy:**	Triticum aestivum

Sequence similarity is available as an NCBI BLAST search of CAI84642.1 against nr.

Search parameters

**Enzyme:**	Trypsin: cuts C-term side of KR unless next residue is P.
**Fixed modifications:**	Carbamidomethyl (C)
**Variable modifications:**	Oxidation (M)
**Mass values searched:**	16
**Mass values matched:**	6

Protein sequence coverage: 55%

Matched peptides shown in ***bold red***.

```
  1 CYPGQAFQVP ALPACRPLLR LQCNGSQVPE AVLRDCCQQL AHISEWCRCG
 51 ALYSMLDSMY KEHGAQEGQA GTGAFPRCRR EVVKLTAASI TAVCRLPIVV
101 DASGDGAYVC KDVAAYPDA
```

Unformatted sequence string: 119 residues (for pasting into other applications).

Sort by (•) residue number ( ) increasing mass ( ) decreasing mass
Show (•) matched peptides only ( ) predicted peptides also

Start – End	Observed	Mr(expt)	Mr(calc)	Delta	M	Peptide
21 – 34	1570.7589	1569.7516	1569.7933	-0.0417	0	R.LQCNGSQVPEAVLR.D
49 – 61	1538.7955	1537.7883	1537.6615	0.1268	0	R.CGALYSMLDSMYK.E
81 – 95	1617.8523	1616.8451	1616.8920	-0.0469	1	R.EVVKLTAASITAVCR.L
85 – 95	1162.5466	1161.5393	1161.6176	-0.0783	0	K.LTAASITAVCR.L
96 – 111	1663.8108	1662.8035	1662.8287	-0.0252	0	R.LPIVVDASGDGAYVCK.D
96 – 119	2466.1834	2465.1761	2465.1785	-0.0024	1	R.LPIVVDASGDGAYVCKDVAAYPDA.-

No match to: 1318.6418, 1372.6236, 1698.8804, 1727.8098, 1766.7635, 1905.9499, 2055.9716, 2211.1040, 2345.1543, 2753.4137

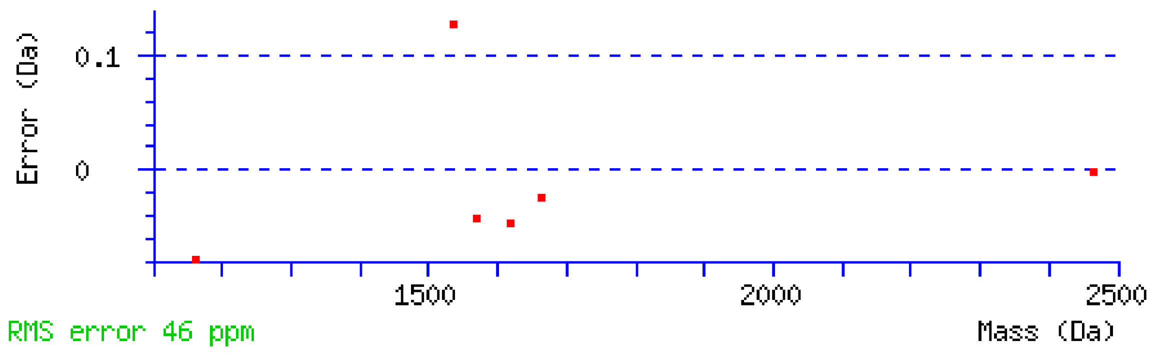

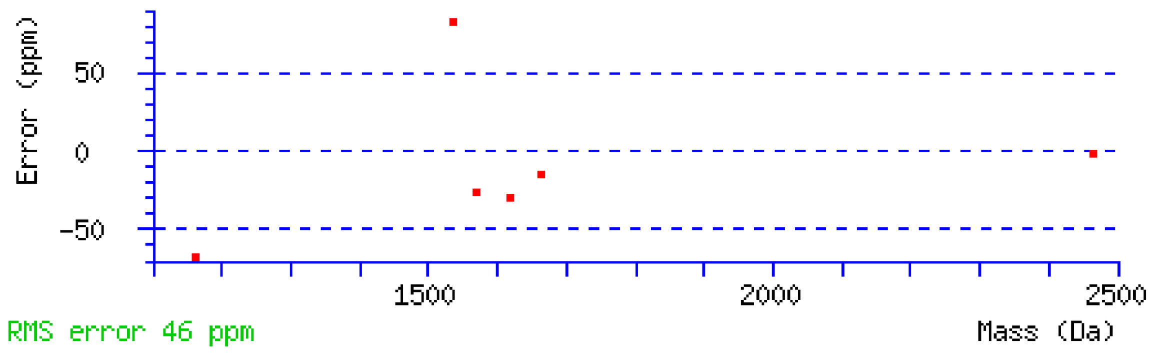

```
LOCUS       CAI84642                 119 aa            linear   PLN 26-JUL-2016
DEFINITION  alpha-amylase inhibitor 0.19, partial [Triticum aestivum].
ACCESSION   CAI84642
VERSION     CAI84642.1
DBSOURCE    embl accession AJ966333.1
KEYWORDS    .
SOURCE      Triticum aestivum (bread wheat)
  ORGANISM  Triticum aestivum
            Eukaryota; Viridiplantae; Streptophyta; Embryophyta; Tracheophyta;
            Spermatophyta; Magnoliophyta; Liliopsida; Poales; Poaceae; BOP
            clade; Pooideae; Triticodae; Triticeae; Triticinae; Triticum.
REFERENCE   1
  AUTHORS   Weichel,M., Vergoossen,N., Bonomi,S., Ballmer-Weber,B.,
            Pastorello,E.A. and Crameri,R.
  TITLE     Cloning cDNA repertoires of IgE-binding proteins of wheat and corn
            screened with sera from food allergic patients that had undergone
            DBPCFC
  JOURNAL   Unpublished
REFERENCE   2  (residues 1 to 119)
  AUTHORS   Weichel,M.
  TITLE     Direct Submission
  JOURNAL   Submitted (21-APR-2005) Weichel M., Molecular Allergology, SIAF,
            Obere Str. 22, CH-7270 Davos Platz, SWITZERLAND
FEATURES             Location/Qualifiers
     source          1..119
                     /organism="Triticum aestivum"
                     /cultivar="Wyuna"
                     /db_xref="taxon:4565"
                     /tissue_type="endosperm"
     Protein         <1..119
                     /product="alpha-amylase inhibitor 0.19"
     Region          1..98
                     /region_name="AAI_SS"
                     /note="AAI_SS: Alpha-Amylase Inhibitors (AAIs) and Seed
                     Storage (SS) Protein subfamily; composed of cereal-type
                     AAIs and SS proteins. They are mainly present in the seeds
                     of a variety of plants. AAIs play an important role in the
                     natural defenses of plants...; cd00261"
                     /db_xref="CDD:238163"
     Site            order(33,85,89,95)
                     /site_type="other"
                     /note="dimer interface [polypeptide binding]"
                     /db_xref="CDD:238163"
     CDS             1..119
                     /gene="0.19"
                     /coded_by="AJ966333.1:<1..360"
                     /experiment="experimental evidence, no additional details
                     recorded"
                     /db_xref="GOA:Q4W0V7"
                     /db_xref="InterPro:IPR003612"
                     /db_xref="InterPro:IPR006105"
                     /db_xref="InterPro:IPR006106"
                     /db_xref="InterPro:IPR013771"
                     /db_xref="InterPro:IPR016140"
                     /db_xref="UniProtKB/TrEMBL:Q4W0V7"
```
