# Supplementary material for: Effects of Germination on Protein, γ-Aminobutyric Acid, Phenolic Acids, and Antioxidant Capacity in Wheat
Source: Molecules. 2018 Sep 3;23(9):2244. doi: 10.3390/molecules23092244 (PMC6225431; doi:10.3390/molecules23092244)
Supplement: Supplementary file 1 [file molecules-23-02244-s001.zip › molecules-331387-supplementary/Peptide list/peptide list_4011.pdf]

MATRIX SCIENCE MASCOT Search Results

Protein View: ABI54569.1

dimeric alpha-amylase inhibitor, partial [Aegilops sharonensis]

**Database:**	NCBIprot
**Score:**	87
**Expect:**	0.012
**Monoisotopic mass (M_r):**	13891
**Calculated pI:**	5.25
**Taxonomy:**	Aegilops sharonensis

This protein sequence matches the following other entries:

- ABI54595.1 from Aegilops longissima
- ABI54596.1 from Aegilops longissima
- ABI54602.1 from Aegilops longissima

Sequence similarity is available as an NCBI BLAST search of ABI54569.1 against nr.

Search parameters

**Enzyme:**	Trypsin: cuts C-term side of KR unless next residue is P.
**Fixed modifications:**	Carbamidomethyl (C)
**Variable modifications:**	Oxidation (M)
**Mass values searched:**	11
**Mass values matched:**	5

Protein sequence coverage: 55%

Matched peptides shown in ***bold red***.

```
  1 SGPWMCYPGQ AFQVPALPGC RPLLRLQCNG SQVPEAVLRD CCQQLADISE
 51 WCRCGALYSM LDDMYKEHGA QEGQAGTGAF PRCRREVVKL TAASITAVCR
101 LPIVVDASGD GAYVCKDVAA YPDA
```

Unformatted sequence string: 124 residues (for pasting into other applications).

Sort by ◉ residue number ○ increasing mass ○ decreasing mass
Show ◉ matched peptides only ○ predicted peptides also

Start – End	Observed	Mr(expt)	Mr(calc)	Delta	M	Peptide
26 – 39	1570.7140	1569.7068	1569.7933	-0.0866	0	R.LQCNGSQVPEAVLR.D
67 – 82	1612.7131	1611.7059	1611.7390	-0.0331	0	K.EHGAQEGQAGTGAFPR.C
86 – 100	1617.8114	1616.8042	1616.8920	-0.0878	1	R.EVVKLTAASITAVCR.L
101 – 116	1663.7980	1662.7907	1662.8287	-0.0380	0	R.LPIVVDASGDGAYVCK.D
101 – 124	2466.2482	2465.2409	2465.1785	0.0625	1	R.LPIVVDASGDGAYVCKDVAAYPDA.-

No match to: 1350.6466, 1372.6162, 1667.7974, 1698.8978, 1715.9218, 2211.1040

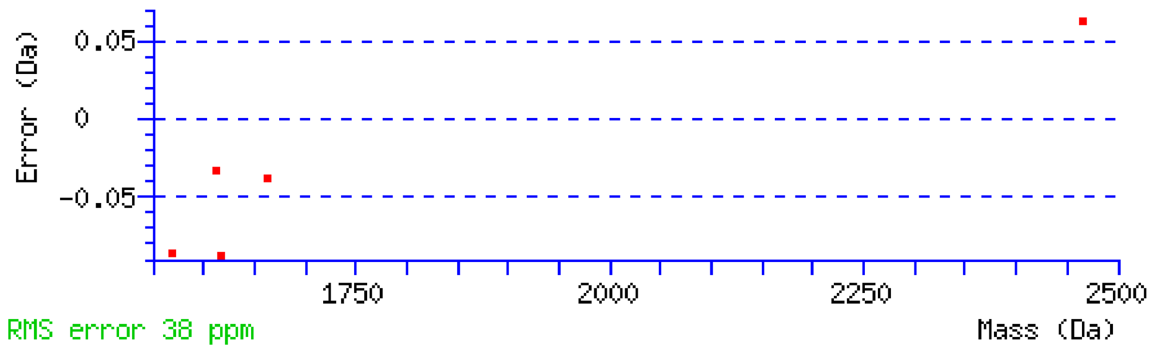

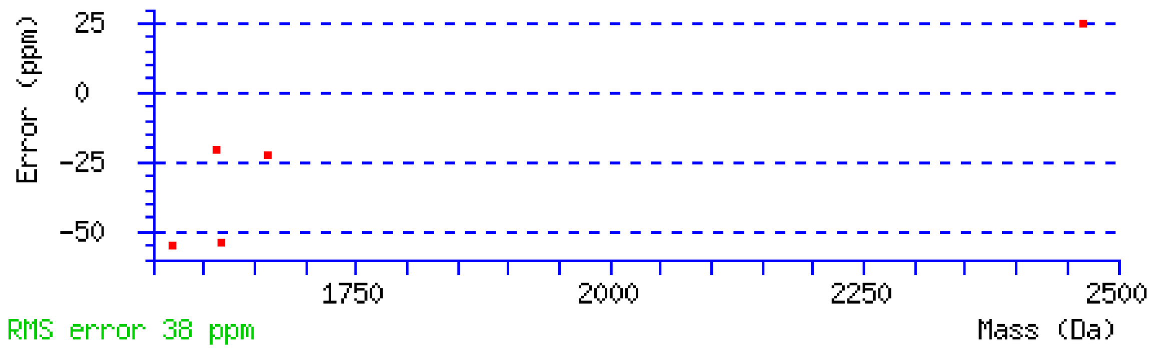


```
LOCUS       ABI54569                 124 aa            linear   PLN 14-JUL-2016
DEFINITION  dimeric alpha-amylase inhibitor, partial [Aegilops sharonensis].
ACCESSION   ABI54569
VERSION     ABI54569.1
DBSOURCE    accession DQ856415.1
KEYWORDS    .
SOURCE      Aegilops sharonensis
  ORGANISM  Aegilops sharonensis
            Eukaryota; Viridiplantae; Streptophyta; Embryophyta; Tracheophyta;
            Spermatophyta; Magnoliophyta; Liliopsida; Poales; Poaceae; BOP
            clade; Pooideae; Triticodae; Triticeae; Triticinae; Aegilops.
REFERENCE   1  (residues 1 to 124)
  AUTHORS   Wang,J.R., Zhang,L., Wei,Y.M., Yan,Z.H., Baum,B.R., Nevo,E. and
            Zheng,Y.L.
  TITLE     Sequence polymorphisms and relationships of dimeric alpha-amylase
            inhibitor genes in the B genomes of Triticum and S genomes of
            Aegilops
  JOURNAL   Plant Sci. 173 (1), 1-11 (2007)
REFERENCE   2  (residues 1 to 124)
  AUTHORS   Wang,J., Wei,Y., Yan,Z. and Zheng,Y.
  TITLE     Direct Submission
  JOURNAL   Submitted (17-JUL-2006) Sichuan Agricultural University, Triticeae
            Research Institute, Yaan Sichuan China, Yaan, Sichuan 625014, China
COMMENT     Method: conceptual translation.
FEATURES             Location/Qualifiers
     source          1..124
                     /organism="Aegilops sharonensis"
                     /cultivar="PI584394"
                     /db_xref="taxon:58530"
                     /clone="5"
     Protein         <1..124
                     /product="dimeric alpha-amylase inhibitor"
     Region          3..103
                     /region_name="AAI_SS"
                     /note="AAI_SS: Alpha-Amylase Inhibitors (AAIs) and Seed
                     Storage (SS) Protein subfamily; composed of cereal-type
                     AAIs and SS proteins. They are mainly present in the seeds
                     of a variety of plants. AAIs play an important role in the
                     natural defenses of plants...; cd00261"
                     /db_xref="CDD:238163"
     Site            order(4,49..51,58)
                     /site_type="other"
                     /note="alpha-amylase binding site [polypeptide binding]"
                     /db_xref="CDD:238163"
     Site            order(38,90,94,100)
                     /site_type="other"
                     /note="dimer interface [polypeptide binding]"
                     /db_xref="CDD:238163"
     CDS             1..124
                     /coded_by="DQ856415.1:<1..375"
```
