# Supplementary material for: Effects of Germination on Protein, γ-Aminobutyric Acid, Phenolic Acids, and Antioxidant Capacity in Wheat
Source: Molecules. 2018 Sep 3;23(9):2244. doi: 10.3390/molecules23092244 (PMC6225431; doi:10.3390/molecules23092244)
Supplement: Supplementary file 1 [file molecules-23-02244-s001.zip › molecules-331387-supplementary/Peptide list/peptide list_4101.pdf]

MATRIX SCIENCE MASCOT Search Results

Protein View: AFG48690.1

hypothetical protein CL3131Contig1_04, partial [Pinus taeda]

**Database:**	NCBIprot
**Score:**	69
**Expect:**	0.68
**Monoisotopic mass (M_r):**	7501
**Calculated pI:**	4.42
**Taxonomy:**	Pinus taeda

This protein sequence matches the following other entries:

- AFG48691.1 from Pinus taeda
- AFG48692.1 from Pinus taeda
- AFG48693.1 from Pinus taeda
- AFG48694.1 from Pinus taeda
- AFG48695.1 from Pinus taeda
- AFG48696.1 from Pinus taeda
- AFG48697.1 from Pinus taeda
- AFG48698.1 from Pinus taeda
- AFG48699.1 from Pinus taeda
- AFG48700.1 from Pinus taeda
- AFG48703.1 from Pinus taeda
- AFG48704.1 from Pinus taeda
- AFG48706.1 from Pinus taeda
- AFG48707.1 from Pinus taeda

Sequence similarity is available as an NCBI BLAST search of AFG48690.1 against nr.

Search parameters

**Enzyme:**	Trypsin: cuts C-term side of KR unless next residue is P.
**Fixed modifications:**	Carbamidomethyl (C)
**Variable modifications:**	Oxidation (M)
**Mass values searched:**	17
**Mass values matched:**	4

Protein sequence coverage: 61%

Matched peptides shown in ***bold red***.

```
 1 EDDAELKAYD RAAMEEGGGD SVWQRGILMG ERCQPPDFSG IILYDVKGNR
51 VPQLPPGSSE LNIFHASL
```

Unformatted sequence string: 68 residues (for pasting into other applications).

Sort by ◉ residue number ○ increasing mass ○ decreasing mass
Show ◉ matched peptides only ○ predicted peptides also

Start - End	Observed	Mr(expt)	Mr(calc)	Delta	M	Peptide
12 - 25	1508.6810	1507.6737	1507.6362	0.0376	0	R.AAMEEGGGDSVWQR.G + Oxidation (M)
12 - 32	2249.0529	2248.0456	2248.0365	0.0091	1	R.AAMEEGGGDSVWQRGILMGER.C
48 - 68	2233.0568	2232.0495	2232.1651	-0.1156	1	K.GNRVPQLPPGSSELNIFHASL.-
51 - 68	1905.9896	1904.9823	1904.9996	-0.0173	0	R.VPQLPPGSSELNIFHASL.-

No match to: 842.3678, 870.4180, 876.9173, 1256.6258, 1428.6688, 1495.7463, 1510.6907, 1570.8308, 1707.7269, 1794.8005, 1851.8923, 2211.1045, 2284.1271

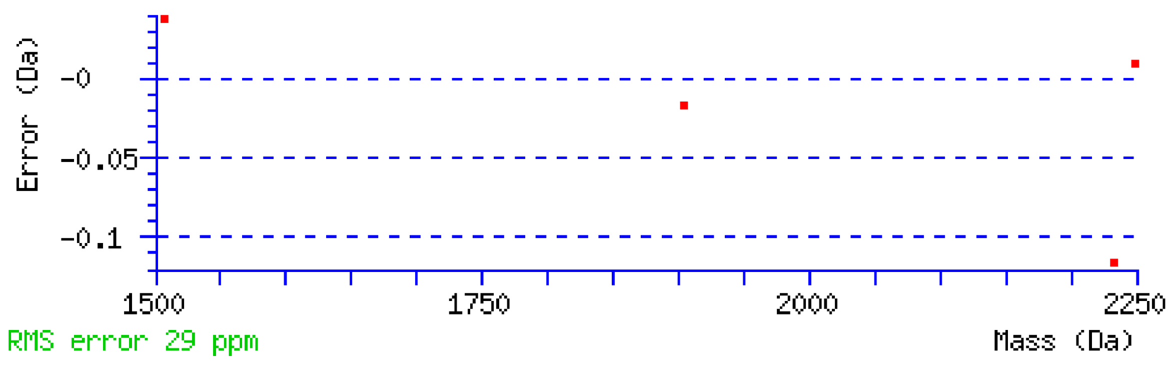

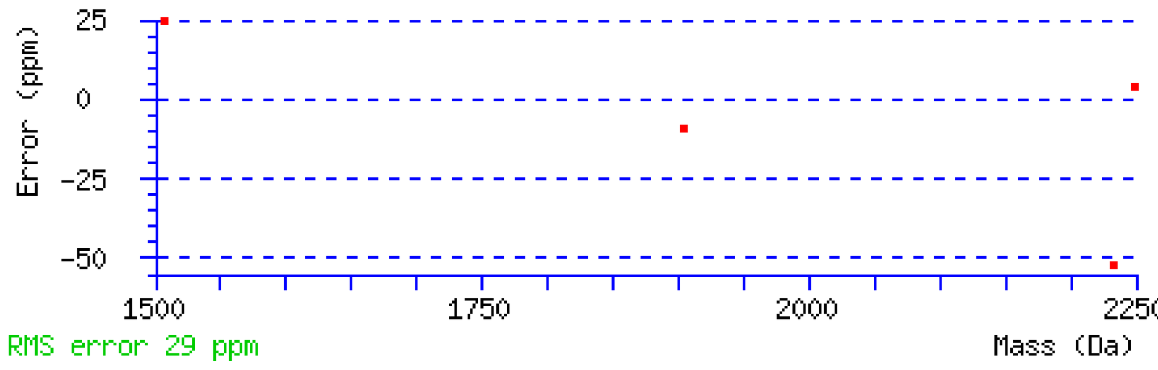

```
LOCUS       AFG48690                  68 aa            linear   PLN 02-APR-2012
DEFINITION  hypothetical protein CL3131Contig1_04, partial [Pinus taeda].
ACCESSION   AFG48690
VERSION     AFG48690.1
DBSOURCE    accession FJ135545.1
KEYWORDS    .
SOURCE      Pinus taeda (loblolly pine)
  ORGANISM  Pinus taeda
            Eukaryota; Viridiplantae; Streptophyta; Embryophyta; Tracheophyta;
            Spermatophyta; Pinidae; Pinales; Pinaceae; Pinus; Pinus.
REFERENCE   1  (residues 1 to 68)
  AUTHORS   Neale,D.B., Wegrzyn,J.L., Lee,J.M., Eckert,A.J., Liechty,J.D.,
            Stevens,K.A. and Langley,C.H.
  TITLE     Nucleotide Diversity and Divergence in the Loblolly Pine Gene Space
  JOURNAL   Unpublished
REFERENCE   2  (residues 1 to 68)
  AUTHORS   Neale,D.B., Wegrzyn,J.L., Lee,J.M., Eckert,A.J., Liechty,J.D.,
            Stevens,K.A. and Langley,C.H.
  TITLE     Direct Submission
  JOURNAL   Submitted (12-AUG-2008) Department of Plant Sciences, University of
            California, Davis, One Shields Avenue, Davis, CA 95616, USA
COMMENT     Method: conceptual translation.
FEATURES             Location/Qualifiers
     source          1..68
                     /organism="Pinus taeda"
                     /isolate="6888"
                     /db_xref="taxon:3352"
                     /tissue_type="megagametophyte"
                     /note="tree id = P.t.10"
     Protein         <1..>68
                     /product="hypothetical protein"
     CDS             1..68
                     /locus_tag="CL3131Contig1_04"
                     /coded_by="FJ135545.1:<1..>207"
                     /note="Similar to XP_002314888.1 [Populus trichocarpa]"
                     /codon_start=3
```

Mascot: http://www.matrixscience.com/
