# Supplementary material for: Effects of Germination on Protein, γ-Aminobutyric Acid, Phenolic Acids, and Antioxidant Capacity in Wheat
Source: Molecules. 2018 Sep 3;23(9):2244. doi: 10.3390/molecules23092244 (PMC6225431; doi:10.3390/molecules23092244)
Supplement: Supplementary file 1 [file molecules-23-02244-s001.zip › molecules-331387-supplementary/Peptide list/peptide list_4201.pdf]

MASCOT Search Results

Protein View: CDJ26374.1

unnamed protein product [Triticum aestivum]

**Database:**	NCBIprot
**Score:**	91
**Expect:**	0.0046
**Monoisotopic mass (M_r):**	21704
**Calculated pI:**	7.81
**Taxonomy:**	Triticum aestivum

Sequence similarity is available as an NCBI BLAST search of CDJ26374.1 against nr.

Search parameters

**Enzyme:**	Trypsin: cuts C-term side of KR unless next residue is P.
**Fixed modifications:**	Carbamidomethyl (C)
**Variable modifications:**	Oxidation (M)
**Mass values searched:**	14
**Mass values matched:**	7

Protein sequence coverage: 35%

Matched peptides shown in ***bold red***.

1 MSTKLLNSLS LRRLLGQQRN PIDLYTASRA WSSSTSFSGV HEKNGMGVEA
51 DGDVADSWKD ADFRGVYR**AI ICGSVGQVPV QK**KLR**NGHIV TVFTVGTGGM**
101 **FDQRR**AGAEN LPMPAQWHR**I AVHNEQLGTY AVQKLVK**NAA VYVEGDIETR
151 VYNDDINNLV KIVPEICVR**F DGKIHLVQSG GSDVSK**SLEE LREGLF

Unformatted sequence string: 196 residues (for pasting into other applications).

Sort by ◉ residue number ○ increasing mass ○ decreasing mass
Show ◉ matched peptides only ○ predicted peptides also

Start – End	Observed	Mr(expt)	Mr(calc)	Delta	M	Peptide
69 – 82	1455.7576	1454.7503	1454.7915	-0.0412	0	R.AIICGSVGQVPVQK.K
86 – 104	2036.0053	2034.9980	2034.9946	0.0034	0	R.NGHIVTVFTVGTGGMFDQR.R
86 – 105	2192.0987	2191.0914	2191.0957	-0.0043	1	R.NGHIVTVFTVGTGGMFDQRR.A
86 – 105	2208.1004	2207.0931	2207.0906	0.0025	1	R.NGHIVTVFTVGTGGMFDQRR.A + Oxidation (M)
120 – 137	2011.0116	2010.0043	2010.1262	-0.1219	1	R.IAVHNEQLGTYAVQKLVK.N
170 – 186	1773.9525	1772.9452	1772.9057	0.0395	1	R.FDGKIHLVQSGGSDVSK.S
174 – 186	1326.6217	1325.6144	1325.6939	-0.0795	0	K.IHLVQSGGSDVSK.S

No match to: 881.1379, 1346.6736, 1516.8052, 1555.6874, 2087.0213, 2095.0477, 2211.1042

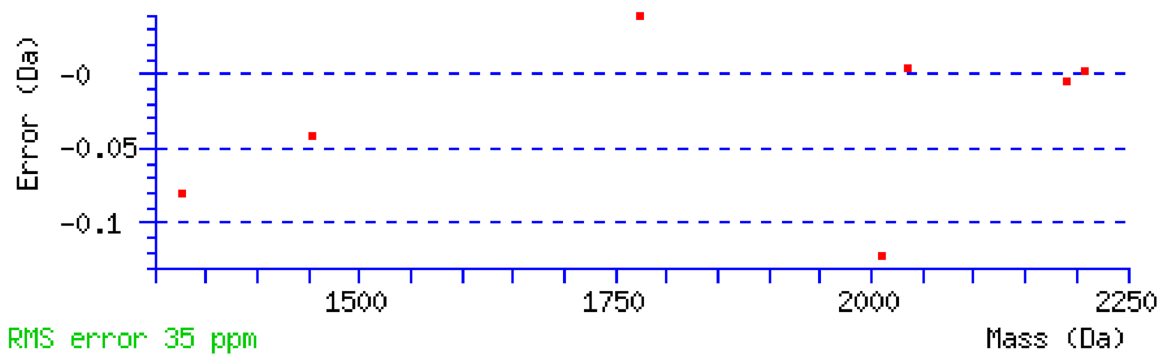

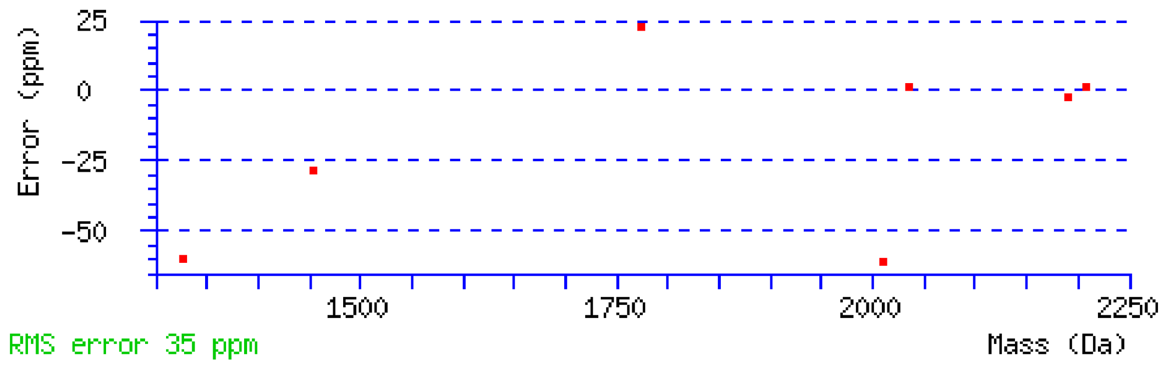


```
LOCUS       CDJ26374                 196 aa            linear   PLN 28-JUL-2014
DEFINITION  unnamed protein product [Triticum aestivum].
ACCESSION   CDJ26374
VERSION     CDJ26374.1
DBLINK      BioProject: PRJEB4376
            BioSample: SAMEA3146289
DBSOURCE    embl accession CBUC010000088.1
KEYWORDS    .
SOURCE      Triticum aestivum (bread wheat)
  ORGANISM  Triticum aestivum
            Eukaryota; Viridiplantae; Streptophyta; Embryophyta; Tracheophyta;
            Spermatophyta; Magnoliophyta; Liliopsida; Poales; Poaceae; BOP
            clade; Pooideae; Triticodae; Triticeae; Triticinae; Triticum.
REFERENCE   1
  AUTHORS   Choulet,F., Alberti,A., Theil,S., Glover,N., Barbe,V., Daron,J.,
            Pingault,L., Sourdille,P., Couloux,A., Paux,E., Leroy,P.,
            Mangenot,S., Guilhot,N., Le Gouis,J., Balfourier,F., Alaux,M.,
            Jamilloux,V., Poulain,J., Durand,C., Bellec,A., Gaspin,C.,
            Safar,J., Dolezel,J., Rogers,J., Vandepoele,K., Aury,J.M.,
            Mayer,K., Berges,H., Quesneville,H., Wincker,P. and Feuillet,C.
  TITLE     Structural and functional partitioning of bread wheat chromosome 3B
  JOURNAL   Science 345 (6194), 1249721 (2014)
   PUBMED   25035497
REFERENCE   2  (residues 1 to 196)
  AUTHORS   Choulet,F.
  TITLE     Direct Submission
  JOURNAL   Submitted (22-AUG-2012) Genetics Diversity and Ecophysiology of
            Cereals, INRA UBP UMR 1095, 234 avenue du Brezet, Clermont-Ferrand,
            F-63100, FRANCE
FEATURES             Location/Qualifiers
     source          1..196
                     /organism="Triticum aestivum"
                     /cultivar="Chinese Spring"
                     /db_xref="taxon:4565"
                     /chromosome="3B"
                     /note="contig v443_0549"
     Protein         1..196
                     /function="conserved_unknown_function - B9FEN7_ORYSJ
                     TrEMBL databank Putative uncharacterized protein OS Oryza
                     sativa subsp japonica GN OsJ_14429 PE 4 SV 1"
                     /name="unnamed protein product"
     CDS             1..196
                     /locus_tag="TRAES_3BF054900050CFD_c1"
                     /coded_by="join(CBUC010000088.1:383185..383251,
                     CBUC010000088.1:383345..383481,
                     CBUC010000088.1:383568..383776,
                     CBUC010000088.1:383902..384002,
                     CBUC010000088.1:384445..384504,
                     CBUC010000088.1:384801..384817)"
                     /note="Low Confidence"
```
