# Supplementary material for: Effects of Germination on Protein, γ-Aminobutyric Acid, Phenolic Acids, and Antioxidant Capacity in Wheat
Source: Molecules. 2018 Sep 3;23(9):2244. doi: 10.3390/molecules23092244 (PMC6225431; doi:10.3390/molecules23092244)
Supplement: Supplementary file 1 [file molecules-23-02244-s001.zip › molecules-331387-supplementary/Peptide list/peptide list_4800.pdf]

# MATRIX SCIENCE MASCOT Search Results

## Protein View: AQT26482.1

**alpha amylase/trypsin inhibitor, partial [Triticum aestivum]**

| | |
|---|---|
| **Database:** | NCBIprot |
| **Score:** | 94 |
| **Expect:** | 0.0021 |
| **Monoisotopic mass ($M_r$):** | 15430 |
| **Calculated pI:** | 7.67 |
| **Taxonomy:** | Triticum aestivum |

Sequence similarity is available as an NCBI BLAST search of AQT26482.1 against nr.

**Search parameters**

| | |
|---|---|
| **Enzyme:** | Trypsin: cuts C-term side of KR unless next residue is P. |
| **Fixed modifications:** | Carbamidomethyl (C) |
| **Variable modifications:** | Oxidation (M) |
| **Mass values searched:** | 8 |
| **Mass values matched:** | 5 |

**Protein sequence coverage: 63%**

Matched peptides shown in ***bold red***.

1 RTNLLPHCR**D YVLQQTCGTF TPGSKLPEWM TSASIYSPGK PYLAK**LYCCQ
51 ELAEISQQCR CEALR**YFIAL PVPSQPVDPR SGNVGESGLI DLPGCPR**QMQ
101 WDFVR**LLVAP GQCNLATIHN VR**YCPAVEQP LWI

Unformatted sequence string: 133 residues (for pasting into other applications).

Sort by ◉ residue number ○ increasing mass ○ decreasing mass
Show ◉ matched peptides only ○ predicted peptides also

| Start - End | Observed | Mr(expt) | Mr(calc) | Delta | M | Peptide |
|---|---|---|---|---|---|---|
| 10 - 25 | 1801.7797 | 1800.7724 | 1800.8353 | -0.0628 | 0 | R.DYVLQQTCGTFTPGSK.L |
| 26 - 45 | 2238.9806 | 2237.9733 | 2238.1394 | -0.1661 | 0 | K.LPEWMTSASIYSPGKPYLAK.L |
| 66 - 80 | 1698.8806 | 1697.8733 | 1697.9141 | -0.0408 | 0 | R.YFIALPVPSQPVDPR.S |
| 81 - 97 | 1727.8040 | 1726.7968 | 1726.8308 | -0.0341 | 0 | R.SGNVGESGLIDLPGCPR.Q |
| 106 - 122 | 1875.9639 | 1874.9567 | 1875.0149 | -0.0582 | 0 | R.LLVAPGQCNLATIHNVR.Y |

**No match to:** 1765.7308, 1794.7927, 2211.1040

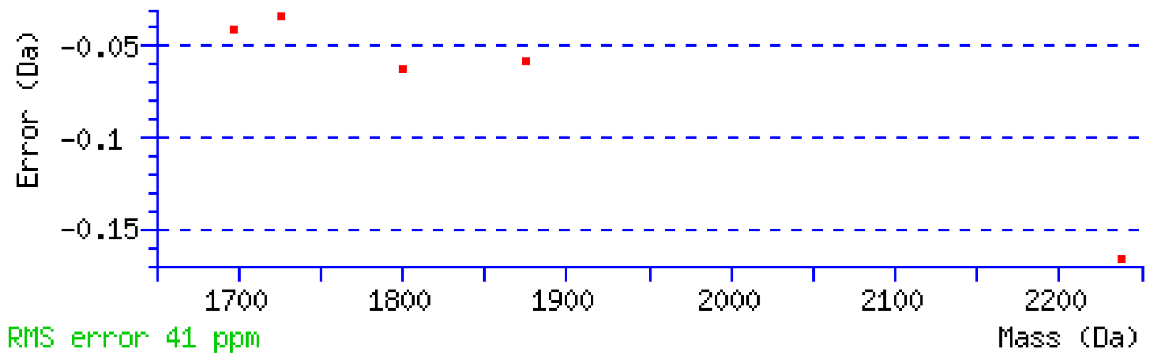


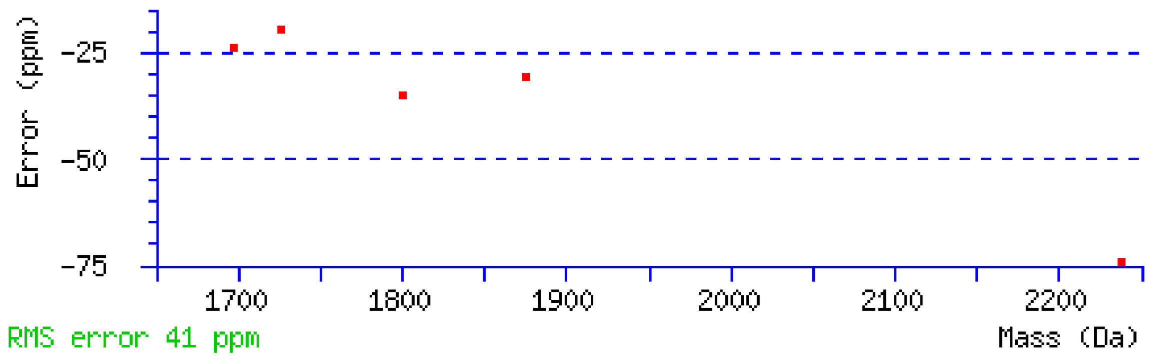


```
LOCUS       AQT26482                 133 aa            linear   PLN 04-MAR-2017
DEFINITION  alpha amylase/trypsin inhibitor, partial [Triticum aestivum].
ACCESSION   AQT26482
VERSION     AQT26482.1
DBSOURCE    accession KY274454.1
KEYWORDS    .
SOURCE      Triticum aestivum (bread wheat)
  ORGANISM  Triticum aestivum
            Eukaryota; Viridiplantae; Streptophyta; Embryophyta; Tracheophyta;
            Spermatophyta; Magnoliophyta; Liliopsida; Poales; Poaceae; BOP
            clade; Pooideae; Triticodae; Triticeae; Triticinae; Triticum.
REFERENCE   1  (residues 1 to 133)
  AUTHORS   Kaushik,M., Sinha,S.K. and Mandal,P.K.
  TITLE     Cloning and sequence analysis of alpha amylase/trypsin inhibitor
            gene from bread wheat (Triticum aestivum)
  JOURNAL   Unpublished
REFERENCE   2  (residues 1 to 133)
  AUTHORS   Kaushik,M., Sinha,S.K. and Mandal,P.K.
  TITLE     Direct Submission
  JOURNAL   Submitted (30-NOV-2016) NRCPB, ICAR-National Research Centre on
            Plant Biotechnology, Pusa Campus, New Delhi, Delhi 110012, India
FEATURES             Location/Qualifiers
     source          1..133
                     /organism="Triticum aestivum"
                     /cultivar="HD2967"
                     /db_xref="taxon:4565"
                     /tissue_type="seed"
                     /country="India"
     Protein         <1..>133
                     /product="alpha amylase/trypsin inhibitor"
     Region          5..125
                     /region_name="AAI_SS"
                     /note="Alpha-Amylase Inhibitors (AAIs) and Seed Storage
                     (SS) Protein subfamily; composed of cereal-type AAIs and
                     SS proteins. They are mainly present in the seeds of a
                     variety of plants. AAIs play an important role in the
                     natural defenses of plants against...; cd00261"
                     /db_xref="CDD:238163"
     Site            order(45,104,108,114,123)
                     /site_type="other"
                     /note="dimer interface [polypeptide binding]"
                     /db_xref="CDD:238163"
     CDS             1..133
                     /gene="ATI"
                     /coded_by="KY274454.1:<1..>403"
                     /codon_start=3
```
