# Supplementary material for: Effects of Germination on Protein, γ-Aminobutyric Acid, Phenolic Acids, and Antioxidant Capacity in Wheat
Source: Molecules. 2018 Sep 3;23(9):2244. doi: 10.3390/molecules23092244 (PMC6225431; doi:10.3390/molecules23092244)
Supplement: Supplementary file 1 [file molecules-23-02244-s001.zip › molecules-331387-supplementary/Peptide list/peptide list_5401.pdf]

# MATRIX SCIENCE MASCOT Search Results

## Protein View: XP_022027330.1

### phospholipase SGR2 isoform X2 [Helianthus annuus]

| | |
|---|---|
| **Database:** | NCBIprot |
| **Score:** | 86 |
| **Expect:** | 0.013 |
| **Monoisotopic mass ($M_r$):** | 100862 |
| **Calculated pI:** | 5.35 |
| **Taxonomy:** | Helianthus annuus |

This protein sequence matches the following other entries:

- XP_022027331.1 from Helianthus annuus

Sequence similarity is available as an NCBI BLAST search of XP_022027330.1 against nr.

### Search parameters

| | |
|---|---|
| **Enzyme:** | Trypsin: cuts C-term side of KR unless next residue is P. |
| **Fixed modifications:** | Carbamidomethyl (C) |
| **Variable modifications:** | Oxidation (M) |
| **Mass values searched:** | 22 |
| **Mass values matched:** | 11 |

### Protein sequence coverage: 17%

Matched peptides shown in ***bold red***.

```
  1 MAESSGKREM ATSADMLRNT PSNIRRLADE IQQCEGRQKY LAQTRSPSDG
 51 GDVRWYFSKI PMAENELAAA IPPTEVVGKG DYFRFGMRDS LAIEASFLQR
101 EEELLSNWWR EYGECSVGPK GSLSSISESN DSPNAQSSKL YANEEERVGV
151 PVKGGLYEVD LLRRHSFPVY WSGDNRRVLR GHWFARKGSL DWFPLREDVA
201 EQLEFAYRSR IWHRRTFQPS GLFAARVDMQ GSSPGLHALF TGEDDTWDAW
251 LNIPSSGFSG VVNFGGTGLK LRRGYAPSHS PKPTQDELRQ QKEEEMDDYC
301 SQVPVRHLVF MVHGIGQRLQ KANLVDDVGT FRLVTQNLAD LHLTSYQRGT
351 QRVLFIPCQW RKNLKLSGES AVEKCTLPGV RGLRITLSAT VHDVLYYMSP
401 IYCQDIIDSV SNQLNRLYMK FLKRNPGYDG KVSLYGHSLG SVLSYDILCH
451 QETLSSPFPM EWMYTDQTHE VSPERNSSVN SPENDLVTNN EGMDTVASHT
501 EGVNGKLSIA TDSEKDDSLV LDEVVVKSGD DPIEMQSENN EVLTSDSSQV
551 DEEINENDES VVIKSLKEEI DCLNARVREL ESQIAEGTNS GTRANKQETS
601 TSRNESVSES FPSSKNDSRK SYTPYIKYTK LLFKVDTFFA VGSPLGVFLA
651 LRNVRIGIGK GKDYWEDENI IEEMPCCRQM FNVFHPHDPV AYRVEPLVSK
701 EYIDKRPVII PYHRGGKRLH VTYQEFVEGV ASRSQVVVDK MSVAKVKVLT
751 ICQSRDNDDH EETSEEPEEI ERSYGSRMLE RLTGSQDGRV DHVLQNKTFE
801 HQYISAVGAH TKYWRDCDTA LFILKHLYRD IPNEPKTSSE DTLSQENADW
851 EELFDNEQLP LTFASEKFMK QLSVRVKKFA NQ
```

Unformatted sequence string: 882 residues (for pasting into other applications).

Sort by (•) residue number ( ) increasing mass ( ) decreasing mass
Show (•) matched peptides only ( ) predicted peptides also

| Start – End | Observed | Mr(expt) | Mr(calc) | Delta | M | Peptide |
|---|---|---|---|---|---|---|
| 27 – 37 | 1318.6559 | 1317.6486 | 1317.5983 | 0.0503 | 0 | R.LADEIQQCEGR.Q |
| 274 – 292 | 2167.0344 | 2166.0272 | 2166.0818 | -0.0546 | 1 | R.GYAPSHSPKPTQDELRQQK.E |
| 307 – 318 | 1393.6438 | 1392.6365 | 1392.7449 | -0.1084 | 0 | R.HLVFMVHGIGQR.L |
| 353 – 362 | 1346.6529 | 1345.6456 | 1345.7329 | -0.0873 | 1 | R.VLFIPCQWRK.N |
| 516 – 527 | 1330.6485 | 1329.6412 | 1329.7028 | -0.0616 | 0 | K.DDSLVLDEVVVK.S |
| 579 – 593 | 1591.7866 | 1590.7793 | 1590.7485 | 0.0308 | 0 | R.ELESQIAEGTNSGTR.A |
| 635 – 652 | 1908.8997 | 1907.8925 | 1908.0509 | -0.1585 | 0 | K.VDTFFAVGSPLGVFLALR.N |
| 734 – 745 | 1306.5803 | 1305.5731 | 1305.6962 | -0.1232 | 1 | R.SQVVVDKMSVAK.V + Oxidation (M) |
| 782 – 797 | 1766.7578 | 1765.7505 | 1765.9071 | -0.1566 | 1 | R.LTGSQDGRVDHVLQNK.T |
| 798 – 815 | 2194.0504 | 2193.0431 | 2193.0756 | -0.0325 | 1 | K.TFEHQYISAVGAHTKYWR.D |
| 826 – 836 | 1381.6547 | 1380.6475 | 1380.7150 | -0.0675 | 1 | K.HLYRDIPNEPK.T |

**No match to:** 870.4201, 1014.4370, 1261.5736, 1276.6542, 1326.6287, 1608.8216, 1646.7789, 2062.8520, 2077.9626, 2162.0488, 2211.1040

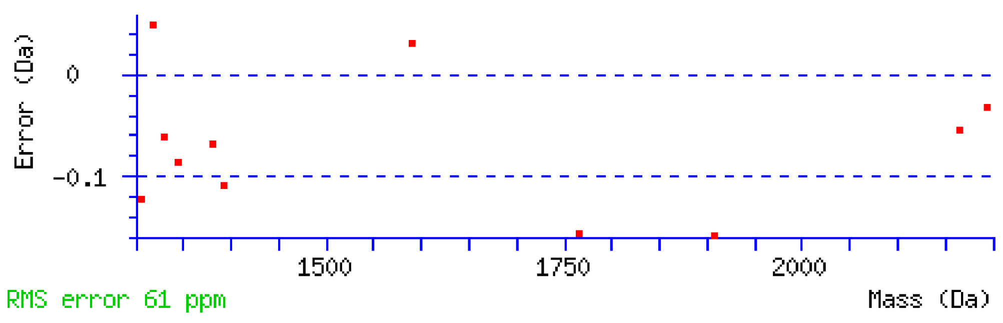


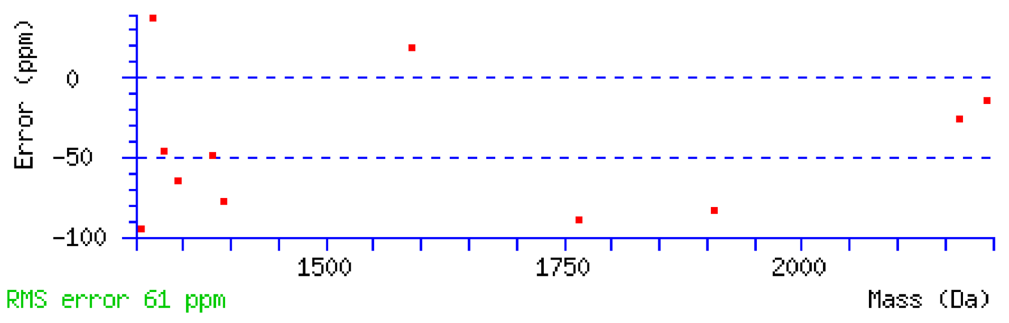


```
LOCUS       XP_022027330             882 aa            linear   PLN 07-AUG-2017
DEFINITION  phospholipase SGR2 isoform X2 [Helianthus annuus].
ACCESSION   XP_022027330
VERSION     XP_022027330.1
DBLINK      BioProject: PRJNA396063
DBSOURCE    REFSEQ: accession XM_022171638.1
KEYWORDS    RefSeq.
SOURCE      Helianthus annuus (common sunflower)
  ORGANISM  Helianthus annuus
            Eukaryota; Viridiplantae; Streptophyta; Embryophyta; Tracheophyta;
            Spermatophyta; Magnoliophyta; eudicotyledons; Gunneridae;
            Pentapetalae; asterids; campanulids; Asterales; Asteraceae;
            Asteroideae; Heliantheae alliance; Heliantheae; Helianthus.
COMMENT     MODEL REFSEQ:  This record is predicted by automated computational
            analysis. This record is derived from a genomic sequence
            (NC_035435.1) annotated using gene prediction method: Gnomon,
            supported by EST evidence.
            Also see:
                Documentation of NCBI's Annotation Process

            ##Genome-Annotation-Data-START##
            Annotation Provider         :: NCBI
            Annotation Status           :: Full annotation
            Annotation Version          :: Helianthus annuus Annotation Release
                                           100
            Annotation Pipeline         :: NCBI eukaryotic genome annotation
                                           pipeline
            Annotation Software Version :: 7.4
            Annotation Method           :: Best-placed RefSeq; Gnomon
            Features Annotated          :: Gene; mRNA; CDS; ncRNA
            ##Genome-Annotation-Data-END##
            COMPLETENESS: full length.
FEATURES             Location/Qualifiers
     source          1..882
                     /organism="Helianthus annuus"
                     /specimen_voucher="SF193"
                     /db_xref="taxon:4232"
                     /tissue_type="leaves"
                     /dev_stage="4 leaves"
                     /country="France"
                     /collected_by="INRA,LIPM"
                     /linkage_group="3"
                     /genotype="XRQ/B"
     Protein         1..882
                     /product="phospholipase SGR2 isoform X2"
                     /calculated_mol_wt=100109
     Region          535..>586
                     /region_name="GrpE"
                     /note="nucleotide exchange factor GrpE; cl03075"
                     /db_xref="CDD:322182"
     Region          634..828
                     /region_name="DDHD"
                     /note="DDHD domain; pfam02862"
                     /db_xref="CDD:308481"
     CDS             1..882
                     /gene="LOC110928610"
                     /coded_by="XM_022171638.1:762..3410"
                     /db_xref="GeneID:110928610"
```
