# Supplementary material for: Effects of Germination on Protein, γ-Aminobutyric Acid, Phenolic Acids, and Antioxidant Capacity in Wheat
Source: Molecules. 2018 Sep 3;23(9):2244. doi: 10.3390/molecules23092244 (PMC6225431; doi:10.3390/molecules23092244)
Supplement: Supplementary file 1 [file molecules-23-02244-s001.zip › molecules-331387-supplementary/Peptide list/peptide list_8009.pdf]

MATRIX SCIENCE MASCOT Search Results

Protein View: AAV39517.1

0.19 dimeric alpha-amylase inhibitor, partial [Triticum aestivum]

**Database:**	NCBIprot
**Score:**	153
**Expect:**	2.9e-09
**Monoisotopic mass (M_r):**	13815
**Calculated pI:**	5.73
**Taxonomy:**	Triticum aestivum

Sequence similarity is available as an NCBI BLAST search of AAV39517.1 against nr.

Search parameters

**Enzyme:**	Trypsin: cuts C-term side of KR unless next residue is P.
**Fixed modifications:**	Carbamidomethyl (C)
**Variable modifications:**	Oxidation (M)
**Mass values searched:**	12
**Mass values matched:**	8

Protein sequence coverage: 92%

Matched peptides shown in ***bold red***.

```
  1 SGPWMCYPGY AFKVPALPGC RPVLKLQCNG SQVPEAVLRE CCQQLADISE
 51 WCRCGALYSM LDSMYKEHGV QEGQAGTGAF PSCRREVVKL TAASITAVCK
101 LPIVIDASGD GAYVCKDVAA YPDA
```

Unformatted sequence string: 124 residues (for pasting into other applications).

Sort by ◉ residue number ○ increasing mass ○ decreasing mass
Show ◉ matched peptides only ○ predicted peptides also

Start – End	Observed	Mr(expt)	Mr(calc)	Delta	M	Peptide
1 – 13	1563.6278	1562.6205	1562.6686	-0.0482	0	-.SGPWMCYPGYAFK.V
14 – 25	1306.7046	1305.6974	1305.7591	-0.0617	0	K.VPALPGCRPVLK.L
26 – 39	1570.7657	1569.7584	1569.7933	-0.0349	0	K.LQCNGSQVPEAVLR.E
40 – 53	1854.7305	1853.7233	1853.7495	-0.0262	0	R.ECCQQLADISEWCR.C
54 – 84	3407.5660	3406.5587	3406.4839	0.0748	1	R.CGALYSMLDSMYKEHGVQEGQAGTGAFPSCR.R
86 – 100	1589.8383	1588.8310	1588.8858	-0.0548	1	R.EVVKLTAASITAVCK.L
90 – 100	1134.5255	1133.5182	1133.6114	-0.0933	0	K.LTAASITAVCK.L
101 – 116	1677.8203	1676.8130	1676.8444	-0.0313	0	K.LPIVIDASGDGAYVCK.D

No match to: 870.4032, 1794.7845, 2211.1040, 2827.3047

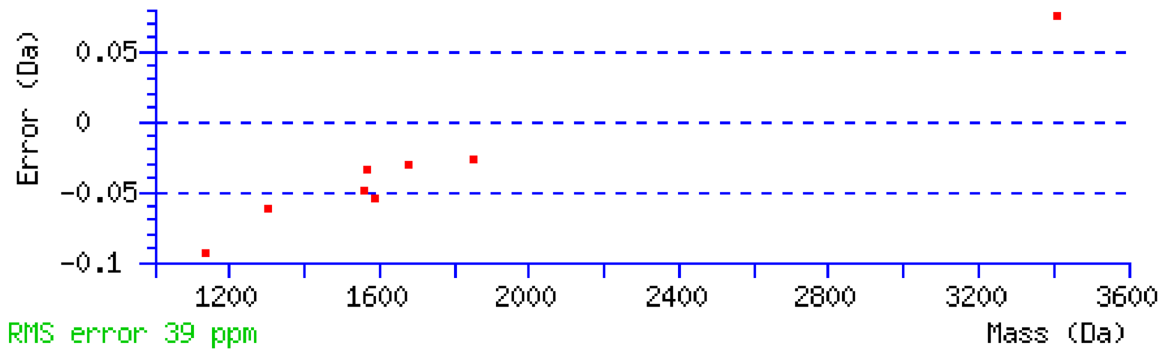

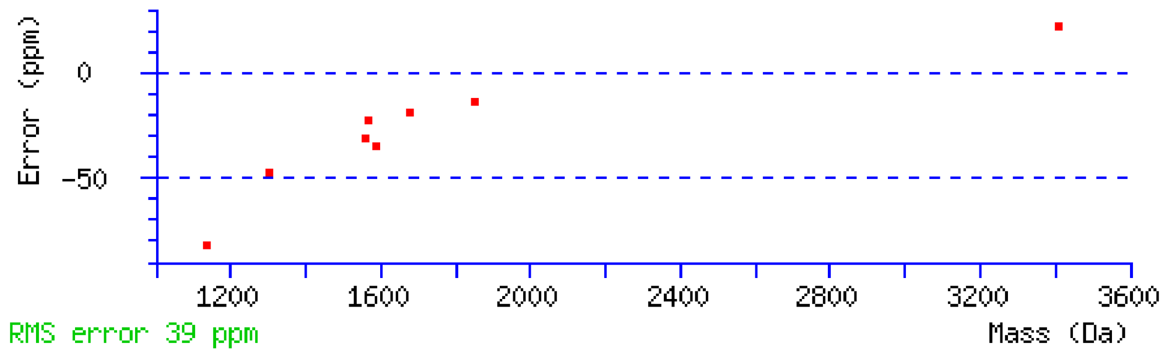


```
LOCUS       AAV39517                 124 aa            linear   PLN 26-JUL-2016
DEFINITION  0.19 dimeric alpha-amylase inhibitor, partial [Triticum aestivum].
ACCESSION   AAV39517
VERSION     AAV39517.1
DBSOURCE    accession AY729675.1
KEYWORDS    .
SOURCE      Triticum aestivum (bread wheat)
  ORGANISM  Triticum aestivum
            Eukaryota; Viridiplantae; Streptophyta; Embryophyta; Tracheophyta;
            Spermatophyta; Magnoliophyta; Liliopsida; Poales; Poaceae; BOP
            clade; Pooideae; Triticodae; Triticeae; Triticinae; Triticum.
REFERENCE   1  (residues 1 to 124)
  AUTHORS   Wang,J.R., Wei,Y.M., Yan,Z.H. and Zheng,Y.L.
  TITLE     Detection of single nucleotide polymorphisms in 24 kDa dimeric
            alpha-amylase inhibitors from cultivated wheat and its diploid
            putative progenitors
  JOURNAL   Biochim. Biophys. Acta 1723 (1-3), 309-320 (2005)
   PUBMED   15837429
REFERENCE   2  (residues 1 to 124)
  AUTHORS   Wang,J., Yan,Z., Wei,Y. and Zheng,Y.
  TITLE     Direct Submission
  JOURNAL   Submitted (14-AUG-2004) Triticeae Research Institute, Sichuan
            Agricultural University, Dujiangyan, Si Chuan 611830, China
COMMENT     Method: conceptual translation.
FEATURES             Location/Qualifiers
     source          1..124
                     /organism="Triticum aestivum"
                     /cultivar="Chuan Nong 16"
                     /specimen_voucher="CDS1367"
                     /db_xref="taxon:4565"
     Protein         <1..124
                     /product="0.19 dimeric alpha-amylase inhibitor"
     Region          3..103
                     /region_name="AAI_SS"
                     /note="AAI_SS: Alpha-Amylase Inhibitors (AAIs) and Seed
                     Storage (SS) Protein subfamily; composed of cereal-type
                     AAIs and SS proteins. They are mainly present in the seeds
                     of a variety of plants. AAIs play an important role in the
                     natural defenses of plants...; cd00261"
                     /db_xref="CDD:238163"
     Site            order(4,49..51,58)
                     /site_type="other"
                     /note="alpha-amylase binding site [polypeptide binding]"
                     /db_xref="CDD:238163"
     Site            order(38,90,94,100)
                     /site_type="other"
                     /note="dimer interface [polypeptide binding]"
                     /db_xref="CDD:238163"
     CDS             1..124
                     /coded_by="AY729675.1:<1..375"
```
