# Supplementary material for: Effects of Germination on Protein, γ-Aminobutyric Acid, Phenolic Acids, and Antioxidant Capacity in Wheat
Source: Molecules. 2018 Sep 3;23(9):2244. doi: 10.3390/molecules23092244 (PMC6225431; doi:10.3390/molecules23092244)
Supplement: Supplementary file 1 [file molecules-23-02244-s001.zip › molecules-331387-supplementary/Peptide list/peptide list_9004.pdf]

MATRIX SCIENCE MASCOT Search Results

Protein View: XP_022553271.1

glutathione S-transferase T3-like [Brassica napus]

**Database:**	NCBIprot
**Score:**	54
**Expect:**	21
**Monoisotopic mass (M_r):**	23636
**Calculated pI:**	8.83
**Taxonomy:**	Brassica napus

Sequence similarity is available as an NCBI BLAST search of XP_022553271.1 against nr.

Search parameters

**Enzyme:**	Trypsin: cuts C-term side of KR unless next residue is P.
**Fixed modifications:**	Carbamidomethyl (C)
**Variable modifications:**	Oxidation (M)
**Mass values searched:**	10
**Mass values matched:**	4

Protein sequence coverage: 22%

Matched peptides shown in ***bold red***.

1 MVNSSTSFIN LLASQGVIDL DSSEPPCVSS QGVASQCSDE ATVKEKRK**WS**
51 **PKEDIILIGA WLNTSKDPIV SNEQK**GLAFW KRIVENYNSS PLLVGTIPRE
101 LGQVKKR**WAR INDLVCKFSG SYEMALR**EQR SGQNDNDVMK AALEIFFNDK
151 GFKFNLEHAW RELRHDVKWC STFLGKDSGK DKRKTGASDA GGSVTEPQER
201 PIGVKAAKAA GG

Unformatted sequence string: 212 residues (for pasting into other applications).

Sort by ◉ residue number ○ increasing mass ○ decreasing mass
Show ◉ matched peptides only ○ predicted peptides also

Start – End	Observed	Mr(expt)	Mr(calc)	Delta	M	Peptide
49 – 66	2070.9956	2069.9883	2070.1149	-0.1266	1	K.WSPKEDIILIGAWLNTSK.D
67 – 75	1029.4253	1028.4180	1028.5138	-0.0958	0	K.DPIVSNEQK.G
108 – 117	1274.6806	1273.6734	1273.6601	0.0132	1	R.WARINDLVCK.F
111 – 127	2002.8759	2001.8687	2001.9652	-0.0965	1	R.INDLVCKFSGSYEMALR.E

No match to: 1306.6755, 1406.7560, 1529.7144, 1819.8732, 1980.9026, 2211.1028

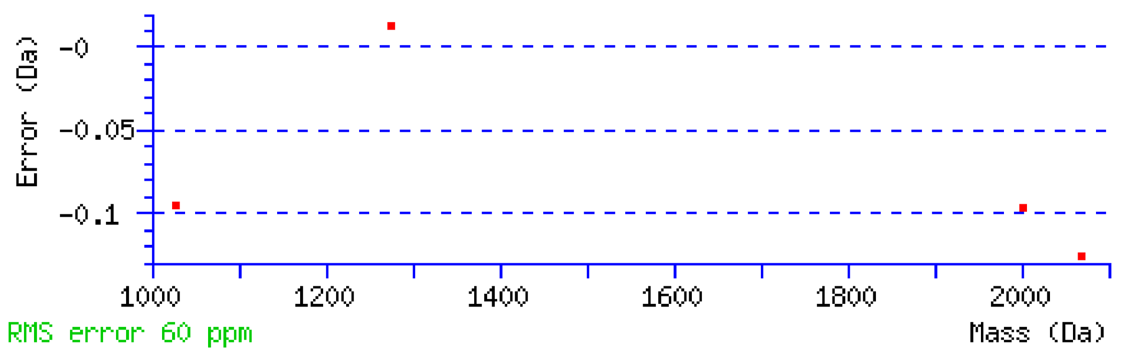

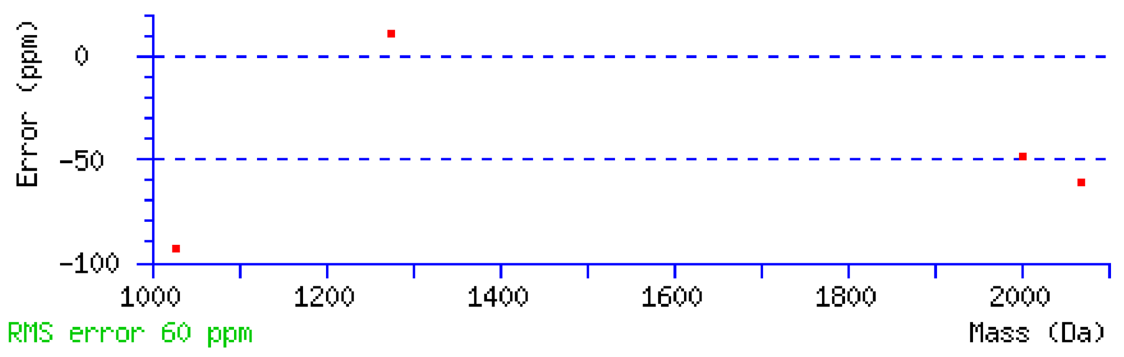


```
LOCUS       XP_022553271             212 aa            linear   PLN 04-OCT-2017
DEFINITION  glutathione S-transferase T3-like [Brassica napus].
ACCESSION   XP_022553271
VERSION     XP_022553271.1
DBLINK      BioProject: PRJNA293435
DBSOURCE    REFSEQ: accession XM_022697550.1
KEYWORDS    RefSeq; includes ab initio.
SOURCE      Brassica napus (rape)
  ORGANISM  Brassica napus
            Eukaryota; Viridiplantae; Streptophyta; Embryophyta; Tracheophyta;
            Spermatophyta; Magnoliophyta; eudicotyledons; Gunneridae;
            Pentapetalae; rosids; malvids; Brassicales; Brassicaceae;
            Brassiceae; Brassica.
COMMENT     MODEL REFSEQ:  This record is predicted by automated computational
            analysis. This record is derived from a genomic sequence
            (NC_027769.2) annotated using gene prediction method: Gnomon.
            Also see:
                Documentation of NCBI's Annotation Process

            ##Genome-Annotation-Data-START##
            Annotation Provider         :: NCBI
            Annotation Status           :: Full annotation
            Annotation Version          :: Brassica napus Annotation Release
                                           101
            Annotation Pipeline         :: NCBI eukaryotic genome annotation
                                           pipeline
            Annotation Software Version :: 7.4
            Annotation Method           :: Best-placed RefSeq; Gnomon
            Features Annotated          :: Gene; mRNA; CDS; ncRNA
            ##Genome-Annotation-Data-END##

            ##RefSeq-Attributes-START##
            ab initio :: 100% of CDS bases
            ##RefSeq-Attributes-END##
            COMPLETENESS: full length.
FEATURES             Location/Qualifiers
     source          1..212
                     /organism="Brassica napus"
                     /cultivar="ZS11"
                     /db_xref="taxon:3708"
                     /chromosome="C3"
     Protein         1..212
                     /product="glutathione S-transferase T3-like"
                     /calculated_mol_wt=23291
     Region          <80..108
                     /region_name="GT1"
                     /note="myb-like, SANT family; cl23759"
                     /db_xref="CDD:329061"
     Region          153..>210
                     /region_name="NAM-associated"
                     /note="No apical meristem-associated C-terminal domain;
                     pfam14303"
                     /db_xref="CDD:316794"
     CDS             1..212
                     /gene="LOC111203635"
                     /coded_by="XM_022697550.1:1..639"
                     /db_xref="GeneID:111203635"
```
